# Phthalate Exposure and Long-Term Epigenomic Consequences: A Review

**DOI:** 10.3389/fgene.2020.00405

**Published:** 2020-05-06

**Authors:** Sudipta Dutta, Diana K. Haggerty, Daniel A. Rappolee, Douglas M. Ruden

**Affiliations:** ^1^Department of Obstetrics and Gynecology, University of Nebraska Medical Center, Omaha, NE, United States; ^2^Department of Epidemiology and Biostatistics, Michigan State University, East Lansing, MI, United States; ^3^Department of Obstetrics and Gynecology, Reproductive Endocrinology and Infertility, CS Mott Center for Human Growth and Development, Wayne State University School of Medicine, Detroit, MI, United States; ^4^Reproductive Stress, Inc., Grosse Pointe Farms, MI, United States; ^5^Institutes for Environmental Health Science, Wayne State University School of Medicine, Detroit, MI, United States

**Keywords:** phthalates, epigenomics, DNA methylation, DOHAD, gestational exposure

## Abstract

Phthalates are esters of phthalic acid which are used in cosmetics and other daily personal care products. They are also used in polyvinyl chloride (PVC) plastics to increase durability and plasticity. Phthalates are not present in plastics by covalent bonds and thus can easily leach into the environment and enter the human body by dermal absorption, ingestion, or inhalation. Several *in vitro* and *in vivo* studies suggest that phthalates can act as endocrine disruptors and cause moderate reproductive and developmental toxicities. Furthermore, phthalates can pass through the placental barrier and affect the developing fetus. Thus, phthalates have ubiquitous presence in food and environment with potential adverse health effects in humans. This review focusses on studies conducted in the field of toxicogenomics of phthalates and discusses possible transgenerational and multigenerational effects caused by phthalate exposure during any point of the life-cycle.

## Introduction

**Figure 1:**
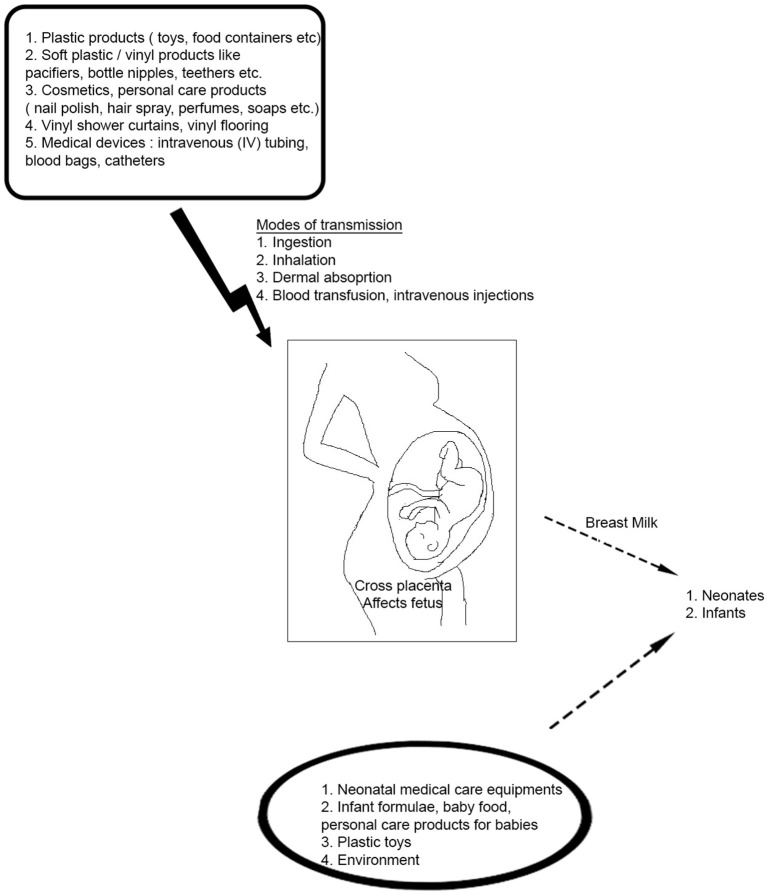
Potential Sources of Phthalate Exposure in daily life. Phthalates have widespread applications in consumer products- they are used in a wide range of daily use household and personal care items starting from soaps, body lotions, and plastic containers to blood transfusion units. They can enter the human body through different routes like ingestion of foods, air inhalation, dust ingestion or dermal absorption. Phthalates can also cross the placenta and affect the developing fetus in a pregnant woman. Infants and neonates are also subjected to phthalate exposure via breast milk and from infant toys like pacifiers, bottle nipples, teethers, and neonatal medical care units.

Phthalates or diesters of phthalic acid are a group of ubiquitous synthetic compounds commonly found in a variety of consumer products like plasticizers since the 1930s (Koch et al., 2013). Historically, the most prevalent one is a high molecular weight (HMW) phthalate, di-(2-ethylhexyl) phthalate (DEHP), which is used in the manufacture of PVC plastics found in items like containers, food packaging, vinyl flooring, furniture, and medical devices (Cirillo et al., 2013). Low molecular weight (LMW) phthalates, including diethyl phthalate (DEP) and butylbenzyl phthalate (BBzP), are used as solvents in the manufacture of daily use personal care products (e.g., perfumes, lotions, cosmetics, shampoo), paints, and adhesives (Buckley et al., 2012). Phthalates are not covalently attached to their substrates and can actively leach into the environment, food, and drinks and thereby enter the human body either through inhalation, digestion, or dermal absorption (Araki et al., 2014; Dewalque et al., 2014) (Figure 1). The persistence of phthalates in the environment makes them an emerging public health concern, as they have potential effects on reproduction, development, obesity, and other public health problems. In animals, phthalates are lipid soluble enabling easy storage in adipose tissue for long periods (Gutierrez-Garcia et al., 2019). In this review, we document several studies that suggest that phthalate exposure in humans, at real-world levels, might have some toxicological risk, with a focus on epigenetic changes associated with phthalate exposures.

### Metabolism of Phthalates

**Figure 2:**
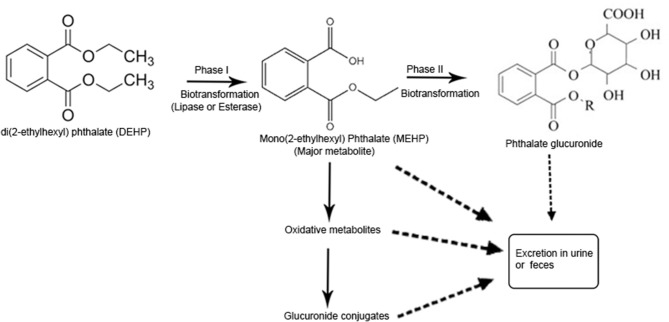
Pathway of phthalate metabolism in human body. LMW phthalates are mainly excreted in urine and feces as a monoester, no further metabolism is required. During phase I hydrolysis, diester phthalates are hydrolyzed by the enzymes like esterases and lipases in the intestine and parenchyma to their respective monoesters. High molecular weight (HMW) phthalates such as diisononyl phthalate (DINP), diisodecyl phthalate (DIDP), and dipropylheptyl phthalate (DPHP) have 9–13 carbon atoms in their chemical backbone and undergo further metabolism from monoesters via hydroxylation or oxidation and produce several oxidative metabolites which are excreted in urine within 24 h of exposure. Oxidative metabolites can also undergo phase II conjugation to form hydrophilic glucuronide conjugates which are excreted. Urinary phthalate metabolite is the most important biomarker for phthalate exposure [Adapted from the article, Metabolism of phthalates in humans by (Frederiksen et al., 2007)].

**Figure 3:**
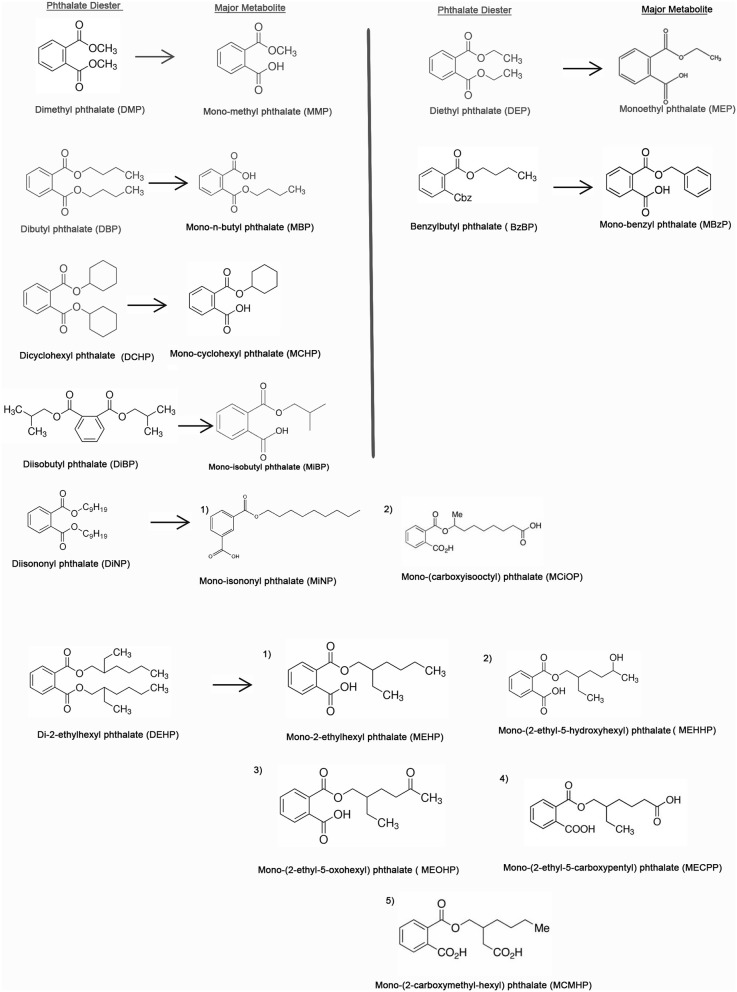
Chemical structures of the top 10 major phthalates and their corresponding metabolites [adapted from the article, A Review of Biomonitoring of Phthalate Exposures by (Wang et al., 2019)].

Phthalates, on human exposure get hydrolyzed to their monoesters and then converted by P450 enzymes to their oxidative metabolites. The metabolites can also be transformed to glucuronide conjugates and released in the urine and feces (ATSDR, 1995, 2001, 2002; Silva et al., 2004). Phthalates which are low molecular weight (LMW) are mostly converted to their monoesters and excreted (ATSDR, 1995, 2001; Silva et al., 2004). DEHP, a commonly found phthalate, is hydrolyzed to its monoester, diethyl phthalate (DEP) and further metabolized in a multi-step pathway to oxidative metabolites which are detected in the urine (ATSDR, 2002). Recent studies suggest that dibutyl phthalate (DBP) and benzylbutyl phthalate (BzBP) are excreted in urine mostly as glucuronidated monoesters like monobutyl phthalate glucuronide (mBP-glu) and monobenzyl phthalate glucuronide (mBzP-glu). DEP is mostly excreted as free mono ethyl phthalate (MEP) (Silva et al., 2003, 2004) and DEHP is excreted as the glucuronidated form of its oxidative metabolites (Kato et al., 2004) (Figure 2). The 10 phthalates most commonly used in consumer products are dimethyl phthalate (DMP), diethyl phthalate (DEP), dibutyl phthalate (DBP), diisobutyl phthalate (DiBP), benzylbutyl phthalate (BzBP), dicyclohexyl phthalate (DCHP), DEHP, di-n-octyl phthalate (DnOP), di-isononyl phthalate (DiNP), and di-isodecyl phthalate (DiDP) (Wang et al., 2019). Some of the major phthalate diesters and their metabolites are shown in Figure 3.

### Overview of Epigenetics

This review focuses on the epigenetic changes associated with phthalate exposure which can potentially be heritable, such as DNA methylation of specific genes in germline cells. DNA methylation of specific genes can alter the expression of these genes without any change of the underlying DNA sequence in cell lineages that differentiate from the germline cells (Das et al., 2008). Epigenetic changes such as DNA methylation are thought to be at the interface of genetics and environment controlling fetal growth and development (Bestor et al., 1988; Okano et al., 1999; Kriaucionis and Heintz, 2009). Genomic DNA methylation occurs at the 5th position of cytosine to give rise to 5-methylcytosine (5mC), in the dinucleotide CpG on both DNA strands. Usually, >70% of CpGs are constitutively methylated in somatic tissues (Wu and Zhang, 2017; Meehan et al., 2018). The cumulative action of DNA methyltransferases (DNMTs) and DNA demethylation pathways help to propagate and maintain the DNA methylation patterns during development creating an unique epigenetic “landscape” to promote genome integrity and maintain cell type specific gene regulatory networks, imprinted gene activity, and repression of transposon activity (Wu and Zhang, 2017; Meehan et al., 2018). DNA methylation reprogramming can take place due to inhibition of DNMTs or *de novo* DNMT activity (Meehan et al., 2018).

In humans and other mammals, it is generally recognized that only stem cells contain the enzymes that can alter the DNA methylation profile of a cell. Embryonic stem cells (ESCs) originate from the inner cell mass at the blastocyst stage of a preimplantation embryo and can differentiate into the three germ layers of the embryo: the ectoderm, endoderm, and the mesoderm (Das et al., 2008; Jeon et al., 2017). While most, if not all cells contain the maintenance DNA methyltrasferase, Dnmt1 (Bestor et al., 1988), it is generally the case that only stem cells contain the “writers” of DNA methylation - Dnmt3a, and Dnmt3b (Okano et al., 1999). Therefore, the putative effects of phthalates on DNA methylation probably involves affecting a writer or an eraser in one of the stem cells in the lineage of the cells being investigated, such as the hematopoietic stem cells from which PBMCs (peripheral blood mononuclear cells) are derived.

Usually, stem cells and a few neuronal cells, contain the “erasers” of DNA methylation known as ten-eleven translocation (TET) family—Tet1, Tet2, and Tet3 (Kriaucionis and Heintz, 2009; Cimmino et al., 2011). Tet1/2 are present in embryonic stem cells and Tet3 is found in the germ line and zygote. All three TET proteins are expressed in blastocysts (Ito et al., 2010). Tet1 preferentially causes promoter demethylation while Tet2/3 act on enhancers (Hon et al., 2014; Huang Y. et al., 2014). TET enzymes participate in DNA methylation dynamics by oxidation of 5mC to 5 hydroxymethylcytosine (5hmC), 5-formylcytosine (5fC), and 5-carboxylcytosine (5caC) as intermediates in DNA demethylation pathways (Meehan et al., 2018). Tet2 is responsible for the vast majority of 5hmC generation (Lio and Rao, 2019). These DNA modifications serve as unique epigenetic signals (Nestor et al., 2012; Meehan et al., 2018). The profiles of 5hmC, 5fC, and 5caC are determined by active gene transcription and enhancer activity. They are less abundant than 5mC but are susceptible to environmental signals and can be used to identify the state of the cell (Meehan et al., 2018).

The most commonly used assays to analyze global DNA methylation levels due to environmental exposures in human cells are the Illumina Human Methylation 450K BeadChip (HM450K) and Illumina Human Methylation 850K BeadChip (EPIC). The HM450K array measures DNA methylation levels at over ~450,000 CpG dinucleotides throughout the genome and has been replaced in 2016 by the EPIC array that measures over >850,000 CpG dinucleotides and and overlaps with about 90% of the sites represented by HM450K chip (Pidsley et al., 2016; Zhou W. et al., 2017). The HM450K and EPIC arrays have been used to study the effects of phthalates on DNA methylation in humans in PBMCs, whole blood, placental tissues, and sperm. In addition to DNA methylation changes induced by phthalates, we also discuss changes in small RNAs such as microRNAs which are thought to be epigenetically transmitted across generations.

## Effect of Phthalates on Different Tissues

### Embryonic Stem Cells (ESCs)

A cost-effective alternative to using laboratory animals in developmental and reproductive toxicity (DART) studies is using embryonic stem cells (ESCs) in so-called embryonic stem cell tests (ESTs). ESCs can make organismal decisions and ultimately give rise to the three main lineages of the embryo—the ectoderm, mesoderm, and endoderm. Human embryonic stem cell (huESC) models serve as *in vitro* models to analyze the epigenetic effects of phthalates on embryonic development since the huESCs form embryoid bodies, which is like an early stage of embryogenesis (Singh and Li, 2012b). *In vitro* differentiation of ESCs is regulated by specific culture conditions. Treatment of culture media with phthalates cause changes in specific gene expression profiles that are predictive of embryotoxicity (van Dartel et al., 2009). Specifically, the top three categories for phthalate toxicity are cardiotoxicity, hepatotoxicity, and nephrotoxicity and the most commonly caused diseases are cardiovascular, liver, urologic, endocrine, and genital diseases (Singh and Li, 2011).

Proper growth and full-term development of the fetus requires a healthy intrauterine environment. In a previous study, a significant relationship was observed between early pregnancy loss (*n* = 48) and elevated periconceptional mono-2-ethylhexyl phthalate (MEHP) exposure (mean: 23.4 ng/mL) (Toft et al., 2012). Since ESCs give rise to all three germ layers-ESCs especially huESCs, serve as an excellent *in vitro* platform to study developmental toxicity of phthalates during pregnancy (Shi et al., 2013). Several labs hypothesize that exposing ESCs to phthalates in culture and determining how this affects their epigenome and differentiation into different lineages might help us to understand the phthalate concentrations which are toxic. In one study, it was revealed that treatment of mouse embryos with a final concentration of 10^−3^ M mono-n-butyl phthalate (MBP) affected the developmental competency, and exposure to 10^−4^ MBP resulted in the delay of progression from embryo to blastocyst (Chu et al., 2013).

Several phthalate esters have been shown to exert developmental toxicity as determined by *in vivo* tests in animals and also by *in vitro* tests such as whole embryo culture (WEC) and embryonic stem cell tests (ESTs) (Shi et al., 2013). In one study (Shi et al., 2013), the authors tested the toxic effects of MEHP on two cell lines of huESCs—CH1, established in their lab with a Chinese female genetic background and H1 with Caucasian male genetic background. MEHP in low concentrations (25 μmol/L or 4,103 ng/mL) after 8 days of treatment in culture did not cause any cytotoxicity but did cause changes the gene expression pattern of several differentiation genes. MEHP in a high concentration (1,000 μmol/L or 164,110 ng/mL) decreased the expression of genes related to mesoderm and the primary germ cells, induced cytotoxicity, and reduced cell proliferation and viability (Shi et al., 2013). Studies examining the effects on *in vitro* expansion of human hematopoietic cells from umbilical cord blood found that four phthalates—DBP, benzyl butyl phthalate (BBP), DEP, and DEHP decreased the cell expansion with DBP being the most cytotoxic (Gutierrez-Garcia et al., 2019).

### Peripheral Blood Mononuclear Cells and Whole Blood Are the Cells-of-Choice to Study the Epigenetic Effects of Environmental Exposures

An ideal *in vitro* research model to study the exposure of xenobiotics in humans is human peripheral blood. After exposure to an environmental toxicant, peripheral blood samples express the hallmarks of epigenetic dysregulation within hours (Baccarelli and Ghosh, 2012). A study (Sicinska, 2019) was conducted to evaluate the effect of DBP, BBP, and their metabolites: MBP, mono-benzylphthalate (MBzP) on apoptosis in human peripheral blood mononuclear cells (PBMC)s after incubation periods of 12 h and/or 24 h. The concentrations tested were in the range of 1 to 100 μg/mL similar to the levels detected in general population exposure (0.02–8 μg/mL) (Chen et al., 2008; Lin et al., 2008; Wan et al., 2013). It was demonstrated that there was a reduction in cell viability after 12 h incubation of PBMCs with phthalates- first by BBP followed by DBP. In the 24 h incubation group, DBP exerted the earliest changes in cell viability, followed by BBP, and by both metabolites (MBP and MBzP) (Sicinska, 2019). To elucidate the mechanism of programmed cell death induced by phthalate exposure, the changes in the level of calcium ions (Ca^2+^), transmembrane mitochondrial potential (ΔΨm) and caspase −8,−9,−3 activity were determined. It was seen that phthalates particularly DBP and BBP increased levels of Ca ^2+^ and reduced ΔΨm of the PBMCs. Phthalates also increased the activity of the caspases, the most significant being caspase−9 (Sicinska, 2019).

A study (Glue et al., 2002) was carried out to estimate the immunotoxicological effects of monophthalates, specifically the cytokine production profiles of MBP, monobenzyl phthalate (MBEP), MEHP, mono-n-octyl phthalate (MOP), mono-isononyl phthalate (MINP), mono-iso-decyl phthalate (MIDP) which are major metabolites of some commonly used phthalates. The monophthalates were used at a concentration of 400 mg/mL. The studies demonstrated that MBP is the only phthalate that lead to an increase in the gene expression of IL-4 with no concomitant increase in the gene expression of IL-5 and IFN- γ. When data was grouped from all phthalate stimulations, there was a significant increase in gene expression of several inflammatory cytokines like IL-4, IL-5, and INF- γ gene (Glue et al., 2002).

Dendritic cells are critical in the development of allergic diseases (von Bubnoff et al., 2001). PBMCs contain the precursors for DC and are frequently exposed to various environmental toxicants. Thus, DCs derived from PBMCs are an excellent model to study immunological responses due to phthalate exposure (Ito et al., 2012). It was seen that only DEHP (not MEHP) at a concentration of 10 μM significantly reduced the expression of markers of maturation and differentiation in DC like CD11c, CD40, CD80, CD86, and CD205 in PBMC-derived DCs of NC/Nga mice. The effects of DEHP on PBMC-derived DCs were partially restored when the cells were treated with an estrogen receptor (ER) antagonist—ICI 182,780 (Ito et al., 2012). Taken together, these findings infer that DEHP attenuated the maturation of PBMC-derived DCs through ER activation.

A study was conducted to compare HM450K and EPIC BeadChips to measure DNA methylation at birth and adolescence. The study used whole blood samples from Mexican-American newborns and 14-year old children (*n* = 109 and *n* = 86, respectively) residing in Salinas Valley, California (Solomon et al., 2018). The overall per-sample correlations analyzed on HM450K and EPIC in both samples was strong (*r* > 0.99), though correlations of individual CpG sites with low variance of methylation were modest (median *r* = 0.24). There was also a subset of CpG sites that had large differences in the mean methylation beta-estimates between the two platforms (Solomon et al., 2018). Finally, the estimates of cell type proportion prediction by the two platforms showed strong correlations in both samples, and differences in boys and girls were successfully replicated across the two platforms (Solomon et al., 2018).

### Placenta

The placenta plays a pivotal role in maintaining the appropriate intrauterine environment by delivering nutrients and oxygen to the developing fetus. It also serves as an important endocrine organ by secreting several hormones plus signaling molecules essential for maintaining the maternal physiology during pregnancy and regulating fetal growth. The placenta is essential to the Developmental Origins of Health and Disease (DOHaD) hypothesis, which posits that *in utero* events program our responses to the environment after birth, and the placenta is important to this process (Gillman et al., 2007; Strakovsky and Schantz, 2018). Any perturbations to the maternal intrauterine environment negatively impacts the long term health status of an infant by acting as a risk factor for adulthood diseases such as cardiovascular disease, obesity, and cancer (Barker, 1997; Nilsson et al., 2012; Zoeller et al., 2012; Radford et al., 2014).

#### Studies of DNA Methylation in Placental Tissue

A recent study investigated how phthalates impair human placental function by epigenetic regulation of critical placental genes (Grindler et al., 2018). The authors looked at epigenome-wide DNA methylation and gene expression using the Agilent whole human genome array and found associations between phthalate exposures during the first trimester of pregnancy and 39 genes with altered DNA methylation and gene expression in the group of women who were highly exposed to phthalates. Further analysis determined epidermal growth factor receptor (EGFR) to be a critical candidate gene that mediates the relationship between exposure to phthalates and early placental function (Grindler et al., 2018). Thus, phthalates may alter the expression of placental genes by epigenetic regulation and thereby affect its regular activity.

The level of phthalates in the third trimester urine of pregnant women was associated with reduced placental long interspersed nucleotide elements (LINE-1) methylation and low birth weight. Placental LINE-1 methylation might serve as a biomarker for environmental exposure causing adverse fetal growth as fetal programming is regulated by appropriate methylation patterning (Zhao et al., 2015).

Genomic imprinting is an epigenetic phenomenon by which genes are methylated to reflect parent of origin expression. The paternally expressed gene Insulin-like growth-factor 2 (IGF2) and maternally expressed H19 both on chromosome 11 are two reciprocally critical imprinted genes and play significant roles in fetal and embryonic growth. Inverse associations were observed between *IGF2* and *H19* differentially methylated regions (DMRs) in placenta and prenatal exposure to HMW phthalate metabolites. Abnormal IGF2/H19 methylation in placenta suggests the fact that the developing fetus may be exposed to an adverse intrauterine environment (LaRocca et al., 2014).

It was demonstrated in at least two studies using human placenta that phthalate exposure during pregnancy was inversely associated with DNA methylation on selected candidate genes like H19 and insulin-like growth-factor 2 (*IGF2*) which are important in embryonic growth and development. These associations were very predominant in fetal growth restriction (FGR) newborns as opposed to normal neonates (LaRocca et al., 2014; Zhao et al., 2016). The placenta persists from the earliest stages of pregnancy through delivery and is responsible for nutrient and gas exchange, waste elimination and thermo-regulation of the developing fetus via mother's circulation. The epigenetic markers in the placenta from an uncomplicated pregnancy/birth vs. a complicated one can thus serve as good indicators of exposures both from intrauterine and extrauterine environments (Rossant and Cross, 2001; Nelissen et al., 2011).

Maternal exposure to phthalates also leads to fetal exposure, as these chemicals can diffuse through the placental barrier and thereby modulate the intrauterine environment (Latini et al., 2003a). In a study designed to investigate the *in utero* effects of human exposure of DEHP and its main metabolite, MEHP, it was observed that phthalate exposure decreased the duration of pregnancy resulting in preterm birth (Latini et al., 2003b). In a sample of 84 newborns, which included 11 preterm births, three very low birthweight newborns, four small-for-gestational-age (SGA) newborns—DEHP, MEHP, or both were found in 88.1% of the cord blood samples, and DEHP and MEHP were individually found in 77.4% of the samples (Latini et al., 2003b). Mean concentrations of DEHP and MEHP in cord blood samples were 1.19 ± 1.15 and 0.52 ± 0.61, μg/mL respectively. Moreover, MEHP-positive newborns showed a significantly decreased gestational age as opposed to MEHP-negative infants (*p* = 0.033) (Latini et al., 2003b).

It has been hypothesized that phthalates may elicit an intrauterine inflammatory response leading to shortened gestation (Goncalves et al., 2002; Latini et al., 2003b). A structural similarity has been observed between DEHP and the proinflammatory mediators like prostaglandins and thromboxanes (Maroziene and Grazuleviciene, 2002). There were also reports of DEHP-induced interleukin-1 secretion in mononuclear cells and in babies born to mothers who suffered from prenatal infection and inflammation (Calo et al., 1993; De Felice et al., 1999, 2002; Yang et al., 2000). Case-control studies to study preterm birth in pregnant women revealed that increased levels of ADAMTS (A Disintegrin and Metalloproteinase with Thrombospondin Motifs) family-ADAMTS4, ADAMTS5 and proinflammatory cytokines like interleukin (IL)-6, and tumor necrosis factor-α (TNF-α) in mid-trimester amniotic fluid associated with spontaneous preterm delivery (Ronzoni et al., 2018; Melekoglu et al., 2019). These data suggest that the toxicity of periconception phthalate exposure and phthalates' capacity to disrupt pregnancy and birth via endocrine and inflammatory pathways impact development and health across the lifespan.

#### Cord Blood

Umbilical cord blood (representing fetal blood) and whole blood samples for 9-year olds were examined to investigate the relationship between *in utero* phthalate exposure and methylation of repetitive elements, Alu and LINE-1. A consistent inverse relationship was observed between prenatal concentrations of MEHP and cord blood methylation of Alu repeats for early and late pregnancy, and a similar association was observed with LINE-1 methylation (Huen et al., 2016). In a longitudinal pregnancy cohort study from California that recruited Mexican-American women and their children, increases in prenatal urinary concentrations of DEHP metabolites gave rise to decreased methylation of Alu repeats. Pyrosequencing of bisulfite-treated DNA was used to analyze the methylation of Alu and LINE-1 (Huen et al., 2016). This observation suggests that prenatal phthalate exposure leads to differences in methylation of repetitive elements and thus epigenetics may be the mechanism by which phthalates exert their transgenerational effect.

A birth-cohort study involving cord blood samples from 64 infant–mother pairs at Taiwan measured DNA methylation levels using the HM450K array was discussed in the introduction section (Chen et al., 2018). Only 25 CpG sites in cord blood with altered methylation levels were significantly associated with DEHP exposure during perinatal period. Gene-set enrichment analysis (GSEA) identified androgen response genes, estrogen response genes, and spermatogenesis genes among the genes enriched via changes in DNA methylation after prenatal DEHP exposure (Chen et al., 2018). Inverse associations were found between maternal phthalate metabolite levels and gestational age, birth weight, birth length, and BMI. Taken together, these studies demonstrate that phthalate exposure *in utero* may impact DNA methylation in cord blood. These changes in DNA methylation may be candidates for biomarkers used to ascertain maternal exposure to phthalates during pregnancy and potential candidates for studying the underlying mechanisms of the long-term effects of phthalates and also the ways phthalates may impact health throughout the life course (Chen et al., 2018).

### Effect of Phthalates as Endocrine Disrupting Chemicals on Embryonic Stem Cells and *in utero*

Phthalates can act as endocrine disrupting chemicals (EDCs) by exerting strong antiandrogenic (Doyle et al., 2013; Martino-Andrade et al., 2016) and weak estrogenic (Lee et al., 2012; Huang P. C. et al., 2014) effects. Though, EDC exposure can be harmful at any stage of human life, the developing human fetus in a phase of rapid proliferative growth *in utero* may be particularly vulnerable (Gutierrez-Garcia et al., 2019). The *in utero* environment or the preconception time period has been regarded as the most vulnerable period of growth and development to environmental insults (Chapin et al., 2004). Additionally, *in utero* phthalate exposure has been associated with pre-term birth (Ferguson et al., 2014), pre-eclampsia (Cantonwine et al., 2016), reduced birth size (Whyatt et al., 2009), sex-specific changes to childhood growth and high blood pressure (Valvi et al., 2015), deficits in neuro-endocrine development (Engel et al., 2010; Kim et al., 2011; Factor-Litvak et al., 2014), and impaired male reproductive health (Cai et al., 2015; Swan et al., 2015). Phthalates can also affect the thyroid hormone balance (Araki et al., 2014) which leads to metabolic dysfunction in adults. Optimal maternal thyroid function during early pregnancy is essential for proper fetal brain development (Chen and Xue, 2018; Ghassabian and Trasande, 2018; Levie et al., 2018; Prezioso et al., 2018).

Phthalates are endocrine disrupting chemicals (EDCs) that mimic the natural hormones found in the human body and thus interfere or impair normal hormonal activity (Grindler et al., 2018). The primary female sex hormones, estrogen (E_2_), and progesterone (P_4_), play important roles in regulating the menstrual cycle, pregnancy, and embryogenesis in humans and other species (Bouman et al., 2005; Hong et al., 2016; Jeon et al., 2017). Apart from that, they also have an effect in regulating the pluripotency of huESCs. E_2_ and P_4_ treatment on human ESCs in a feeder-free culture protocol decreases the pluripotency of human ESCs by inhibiting the expression of pluripotency-associated markers like POU class 5 homeobox 1 (*POU5F1), sex determining region Y-box 2 (SOX2)*, and *NANOG* homeobox genes at both transcriptional and translational levels. The cells growing in control culture media without any hormones assumed the form of tightly packed cells growing in a monolayer with clean and defined edges and showed no signs of differentiation. These cells also expressed several markers specific for undifferentiated ES cells including POU5F1, SOX2, and NANOG (Jeon et al., 2017).

The female sex hormones E_2_ and P_4_ also alter the protein expression of markers for the epithelial-to-mesenchymal transition (EMT). E_2_ or P_4_ treatment increases the protein expression levels of N-cadherin, Snail and Slug, which are highly expressed in mesenchymal cells and decreases E-cadherin expression, which is highly expressed in epithelial cells. Phthalates exert weak estrogenic effects (Lee et al., 2012; Huang P. C. et al., 2014) and thus they can perturb normal pregnancy by decreasing the pluripotency of the stem cells in developing pre-implantation embryos (Jeon et al., 2017). Further validation studies involving treatment of E_2_ and P_4_ in combination with estrogen and progesterone receptor inhibitors (ICI 182,780 and RU486 respectively) found the effects of hormones on EMT and pluripotency of ES cells were restored to control levels. These findings indicate that E_2_ and P_4_ regulation of EMT and pluripotency of human ES cells are mediated by their receptors (Jeon et al., 2017). Bone marrow (BM) hematopoietic stem progenitor cells express functional receptors for follicle-stimulating hormone (FSH), and luteinizing hormone (LH). *In vitro and in vivo*, pituitary sex hormones such as FSH, LH, and prolactin (PRL) stimulate hematopoietic stem progenitor cells to proliferate. These cells also proliferate in response to gonadal sex hormones like androgen, estrogen, and progesterone (Carreras et al., 2008; Maggio et al., 2013; Nakada et al., 2014; Mierzejewska et al., 2015). These data elicit an interesting avenue where phthalates can interrupt normal embryonic growth and development by impairing the pluripotency of ESCs and by causing a misregulation of the EMT.

### Germ Cells (Eggs and Sperm) and Ano-Genital Distance

#### Epigenetic Changes in Sperm

Current research findings suggest that several adulthood diseases are programmed *in utero* as a result of maternal exposures to endocrine disruptors like DEHP. Those diseases are linked with critical genes which are epigenetically modified by DNA methylation or modification of histone tails (Martinez-Arguelles et al., 2009; Wu et al., 2010; Anderson et al., 2012; Strakovsky and Pan, 2012; Ayala-Garcia et al., 2013). The first reprogramming event occurs in the genome of the germ cell precursors while they colonize the embryo's urogenital crest. This imprinting event creates an epigenetic memory on the gamete's genome that represents the environment around them while they are committed to the gamete lineage (Jaenisch et al., 2004; Surani et al., 2004). In sexually mature males, a second round of gamete epigenetic reprogramming occurs during differentiation to give rise to spermatozoa. The spermatogonial populations can constantly reedit the epigenetic information, which enables them to inherit an updated epigenetic print that reflects the varying environmental situations. It is hypothesized that such epigenetic reprogramming permits the spermatozoa of several generations to provide updated information about the environment during consecutive periods of fertilization and transmit this information to the offspring. The next event of epigenetic reprogramming of both the paternal and maternal chromosomes occurs shortly after fertilization. Thus, experiments which involve prenatal and postnatal exposure to phthalates may unravel mechanism underlying the epigenetic modulation of gene expression for phenotypic variability between individuals and across species (Ayala-Garcia et al., 2013).

One study in rats reports that *in utero* exposure to DEHP impaired testicular function through changes in DNA methylation (Sekaran and Jagadeesan, 2015). It was observed in a separate study that *in utero* exposure to DEHP in rats was associated with both transgenerational DNA methylation in sperm and testicular and prostate diseases (Manikkam et al., 2013), while another study in rats reported that *in utero* exposure to DEHP alters DNA methylation throughout the epigenome, particularly in CpG islands (Martinez-Arguelles and Papadopoulos, 2015).

Various animal studies have established the fact that *in utero* phthalate exposure gives rise to transgenerationally inherited reproductive defects by altering sperm DNA methylation (Manikkam et al., 2013; Iqbal et al., 2015; Prados et al., 2015). To address the relevance of epigenetic reprogramming of sperm in humans, a study was conducted to examine the relationship of pre-conception urinary phthalate with sperm DNA methylation profiles in men undergoing fertility treatment in IVF clinics (Wu et al., 2017a). In a study performed by HM450K analyses, 131 sperm DMRs were correlated with at least one preconception urinary metabolite. The DMRs were typically clustered with genes responsible for growth and development and other functions like cellular movement and cytoskeleton structure. Most sperm DMRs were associated with phthalate metabolites like MEHP, mono (2-ethyl-5-oxohexyl) phthalate (MEOHP), MBP and cyclohexane-1, 2-dicarboxylic acid-monocarboxy isooctyl (MCOCH), which are anti-androgenic. Furthermore, 13% of sperm DMRs could determine reproductive success and were attributable for diminished quality blastocyst-stage embryos after *in vitro* fertilization (IVF) (Wu et al., 2017a,b).

#### Ano-Genital Distance

The anogenital index (AGI) is defined as anogenital distance (AGD) divided by weight at examination [AGI = AGD/weight (mm/kg)]. The rationale for measuring AGD is that males have a shorter AGD than females and consequently, changes in this distance are a measure of feminization or masculinization of the reproductive organs. In a study that aimed to correlate prenatal exposure to phthalates in amniotic fluid, maternal urine, and health of newborns in humans, it was found that *in utero* exposure to MBP was associated with a shortened AGI in female newborns, although no correlation was found between prenatal phthalate exposure *in utero* and AGI in male newborns (Huang et al., 2009). In a study that involved analysis of 106 boys aged around 12.8 months in the population, genital measurements (including AGD) were measured in relation to concentrations of phthalate metabolites in maternal prenatal urine samples. AGD was significantly and inversely related to maternal urinary concentrations of metabolites of DEHP. Incomplete testicular descent was also observed in those boys (Swan et al., 2005; Swan, 2008). These findings demonstrate that phthalate exposure may disrupt human male genital development. Females may experience reproductive toxicity due to phthalates as severe as that observed in males (Benjamin et al., 2017). Recent reports suggest that high urinary phthalate concentrations were linked with delayed attainment of puberty in girls (Frederiksen et al., 2012), increased endometriosis risk (Cobellis et al., 2003; Masuyama et al., 2003; Upson et al., 2013), low yield of oocytes (Hauser et al., 2016), increased incidences of infertility (Du et al., 2016), and increased clinical pregnancy loss (Mu et al., 2015; Hauser et al., 2016). To model some of these effects of phthalates seen in humans in mammalian models, there are several reports that prenatal phthalate exposure affects testicular function and is responsible for decreasing anogenital distance (AGD) in male rodents (Mylchreest et al., 1998; Wolf et al., 1999; Gray et al., 2000; Ema et al., 2003; Tyl et al., 2004; Carruthers and Foster, 2005; Andrade et al., 2006).

## Phthalate Exposure Across the Lifespan

### Phthalate Exposure in Neonatal Intensive Care Units

DEHP is primarily used to soften PVC plastic in medical apparatus like blood bags, or bags used for intravenous administration of nutrients, drugs, and fluids. DEHP can leach out from the plastic that is used to make all of these bags to enter the patient's circulation during procedures like transfusion, heart bypass surgery, or the administration of intravenous fluids (Latini, 2000; Tickner et al., 2001). Based on a report by the Center for Devices and Radiological Health, U.S. Food and Drug Administration, devices used in Neonatal Intensive Care Units (NICU) are a prime concern because newborns undergoing procedures using these medical devices may be exposed to DEHP levels ranging from 130 to 6,000 μg/kg bw/day (Hillman et al., 1975; Plonait et al., 1993; Latini and Avery, 1999; Latini, 2000; Loff et al., 2000; Food U. S. Drug Administration, 2001; Tickner et al., 2001). The NICU babies are exposed to plastic tubing, blood bags, IV drips, etc. Particularly in a NICU setting, neonates would be small for two main reasons: (i) they might be preterm which makes them very underweight since they were born early; (ii) neonates who had intrauterine growth restriction (IUGR) develop many health complications and may be small for their gestational age (SGA). Children, due to their low body weights and their underdeveloped organs, are at a higher risk than adults to phthalate exposure. In fact, people of all ages who undergo such medical procedures are exposed to fairly high levels of DEHP (Shelby, 2006).

### Asthma

An epidemiological study linked phthalate exposure to lower DNA methylation of TNFα which is an inflammatory cytokine that may increase asthma risk in children (Wang et al., 2015). Differential methylation patterns have been observed in three genes-*androgen receptor* (*AR*), *TNF*α, and *IL*-*4* causing asthma in children (Wang et al., 2015). TNFα 5'CGI is a potential epigenetic biomarker for uncovering a phthalate mechanism in childhood asthma research. Hypomethylation of TNFα 5'CGI gives rise to increased TNFα protein levels which may give rise to allergic inflammation (Wang et al., 2015). TNFα is present in high concentrations in bronchoalveolar fluid derived from the airways of asthma patients (Broide et al., 1992; Berry et al., 2006). A study involving a transgenerational asthma model in mouse demonstrated that maternal exposure to BBP could cause allergic airway inflammation in the offspring over 2 generations (F2). In the offspring, BBP induced global DNA hypermethylation in CD^4+^ T cells (Jahreis et al., 2018).

### Lipid Metabolism

It was observed that exposure to DEHP *in utero* in pregnant mice induces excessive visceral fat accumulation, affects lipid metabolism and adipogenesis in their F1 offspring (Gu et al., 2016). The study was conducted with pregnant C57BL/6J mice who were administered with DEHP (0.05 mg/kg/day) from gestational days 1-19, the pups had significantly higher levels of serum leptin, insulin, lipid, and fasting glucose concentrations than the control pups. DEHP-exposed pups also had excessive visceral fat accumulation compared to control pups. These metabolic disorders were hypothesized to be induced by elevated levels of mRNA expression of T-box 15 (*Tbx15*) and glypican 4 (*Gpc4*) in subcutaneous and visceral adipose tissues respectively. *Tbx15 and Gpc4* are known to be developmental genes which play a role in obesity and body fat distribution in mice (Gesta et al., 2006; Gu et al., 2016).

In a longitudinal study involving 250 Mexican American children, the relationship between perinatal exposure to phthalates and adiposity during the peri-adolescence (between ages 8 and 14 years) was evaluated (Bowman et al., 2019). Among girls, adiposity was associated with exposure to MBP, MiBP, and MBzP. First trimester maternal urine concentrations of MiBP were associated with increased values for skinfold thickness, BMI-for-age, and waist circumference in girls (*p* < 0.01) as opposed to control samples. *H19* methylation was positively associated with skinfold thickness in girls. There were sex-specific differences in exposure outcomes- among boys, adiposity was inversely associated with second trimester and adolescent MBzP (Bowman et al., 2019).

Lipid metabolism is important for synthesis of steroid hormones and phthalate-driven aberrant lipid metabolism disrupts the normal metabolic and reproductive processes (Moody et al., 2019). In this study (Moody et al., 2019), it was demonstrated that when pregnant Long-Evans rats were administered mixture of phthalates 0 (CON), 200 (LO), or 1,000 (HI) mg/kg body weight/day during the perinatal period-the male offspring for both groups at PND90 had higher body weights than control. Sterol regulatory element binding proteins (SREBPs) have been hypothesized to play a pivotal role in phthalate-induced metabolic dysregulation (Johnson et al., 2011; Zhang et al., 2017). In both testis and adipose tissue of males belonging to the HI phthalate dosage, gene expression of lipid metabolism pathways were dysregulated. *Srebf1* expression was reduced in testis whereas *Srebf2* was upregulated in adipose tissue. DNA methylation was increased at two loci in testis of HI rats and reduced at another site surrounding Srebf1 transcription start site. Simultaneously, in rats belonging to the HI phthalate dosage group- in the adipose tissue increased DNA methylation at one region was observed within the first intron of *Srebf2* (Moody et al., 2019). Thus, phthalate exposure impairs metabolism of lipids by DNA methylation through tissue-specific changes in gene expression.

### Adulthood Diseases

#### Obesity

Phthalates are associated with a number of diseases in adults due to their endocrine-disrupting abilities (Gore et al., 2015). One of the common disorders associated with EDCs is obesity in children and adults (Biemann et al., 2014; Di Ciaula and Portincasa, 2019). Obesity is recognized as a public health epidemic in both developed and developing countries. As per convention, for adults (individuals above the age of 18 years), overweight is defined as having a body-mass index (BMI) greater than or equal to 25 and lower than 30 and obesity is defined as having a BMI greater than or equal to 30 (Ng et al., 2014). A statistic from 188 countries indicates that between 1980 and 2013, combined percentage of overweight and obesity has increased by 27.5% for adults and 47.1% for children (Ng et al., 2014). For both developed and developing countries, the proportion of adults with a BMI of 25 or greater increased from 28.8% in 1980 to 36.9% in 2013 for men and from 29.8% to 38% for women (Ng et al., 2014). The peroxisome proliferator-activated receptor (PPAR)⋎, a nuclear receptor is regarded as the master regulator of adipogenesis and regulates the expression of metabolic genes during differentiation (Janesick and Blumberg, 2011; Stel and Legler, 2015).

Obesogenic EDCs have the ability to stimulate adipogenesis and fat storage and increase the chances for obesity by activating PPAR⋎ (Stel and Legler, 2015). PPAR⋎ acts on the differentiation pathway connecting multipotent stromal stem cells to mature adipocytes (Janesick and Blumberg, 2011; Watt and Schlezinger, 2015). PPAR⋎ regulates histone deacetylation, changes in DNA methylation and modulates a series of mechanistic pathways leading to increase in adipocyte formation and fat storage (Tabb and Blumberg, 2006; Blumberg, 2011; Janesick and Blumberg, 2012; Rajesh and Balasubramanian, 2014; Stel and Legler, 2015; Watt and Schlezinger, 2015). One study using a primary mouse bone marrow culture model demonstrated that phthalates interact with PPARs and regulate the expression of genes involved in adipocyte differentiation, adipogenesis, and metabolic processes like lipid and glucose homeostasis (Desvergne et al., 2009; Grygiel-Gorniak, 2014; Watt and Schlezinger, 2015). The Developmental Origins of Health and Disease (DOHaD) hypothesis is a paradigm in which prenatal and perinatal exposure to environmental factors plays a pivotal role in determining life-long patterns of health and disease (Gluckman and Hanson, 2004). The Newcastle thousand families study, which consisted of 932 members of thousand families 1947 birth cohort, aimed to track whether being overweight in childhood increases the risk of adult obesity (Wright et al., 2001). The study examined 412 subjects at age 50 and found that BMI at age 9 years was significantly correlated with BMI at age 50 and only children who were obese at 13 had an increased risk of obesity during adulthood (Wright et al., 2001). Maternal urinary levels of mono-3-carboxypropyl phthalate (MCPP), a non-specific metabolite of multiple phthalates, caused childhood obesity in a study that recruited 707 children from three prospective cohort studies in the USA between 1998 and 2006 (Buckley et al., 2016). The study explored the relationship between maternal urinary phthalate metabolite concentrations during pregnancy with weight and height of children at ages 4 to 7 years (Buckley et al., 2016). Metabolites of DEP and DEHP were associated with sexually dimorphic effects on BMI and ΣDEHP was inversely related with BMI z-scores among girls, but no association was noted in boys (Buckley et al., 2016). Children's Health and Environmental Chemicals in Korea (CHECK) Study recruited 128 healthy pregnant women and their newborns (65 boys and 63 girls)—levels of DEHP metabolites were measured in maternal blood, urine, placenta, and cord blood samples as well as newborns' urine (Kim et al., 2016). The study revealed that DEHP exposure may decrease ponderal index (PI) and increase triglyceride (TG) levels in newborn infants especially boys (PI, β = −0.13, *p* = 0.021; and TG, β = 0.19, *p* = 0.025) causing increase in body mass in early life. This observation also suggested that *in utero* exposure to DEP and DEHP was positively associated with body mass change of the newborns during first 3 months after birth (Kim et al., 2016).

#### Men's Reproductive Health

The major male reproductive anomaly associated with phthalates is “testicular dysgenesis syndrome” which is characterized by hypospadias, cryptorchidism, undescended testes, reduced anogenital distance, reduction in sperm count and quality, sterility, and occurrence of testicular cancer (Sharpe and Skakkebaek, 2008; Swan, 2008). Anogenital distance (distance between anus and genitalia) is the most sensitive marker for estimating the impact of phthalates in human males; this anomaly is associated with prenatal exposure of the male fetus to phthalates while in the womb (Swan et al., 2005; Marsee et al., 2006; Suzuki et al., 2012). Phthalates bind to histone tails thus regulating the extent of DNA enclosed by it and thereby alter the availability of genes which can be activated (Wu et al., 2010; Manikkam et al., 2013). One study (Wu et al., 2010) showed that in mice, maternal exposure of DEHP caused testicular dysfunction which was mediated by DNA hypermethylation, leading to increased expression of DNA methyltransferases and downregulated production of insulin like hormone-3, a gene responsible for testosterone production. Phthalate monoesters like mono-n-butyl phthalate (mBP), mono-ethyl phthalate (mEP) found in human breast milk have a positive correlation with postnatal surge of hormones like serum hormone binding globulin (SHBG) in newborn boys. Phthalates like mono-methyl phthalate (mMP), mono-ethyl phthalate (mEP), and mono-n-butyl phthalate (mBP) were directly related with the ratio of LH:free testosterone and mono-isononyl phthalate (miNP) with luteinizing hormone (LH). mBP was negatively correlated with free testosterone and these hormonal imbalances could be a sign of testicular dysgenesis (Main et al., 2006). Testicular dysgenesis syndrome can lead to impaired spermatogenesis and is associated with testicular cancer in adult men (Virtanen et al., 2007).

#### Allergies and Asthma

High molecular weight phthalates like DEHP, BBP, and their monoesters have been associated with allergies, asthma, wheezing, hay fever, itchy rashes, and eczema in adults. These phthalates are hypothesized to affect disease of the airways through increased levels of oxidative stress and secretion of several inflammatory cytokines like IL-4, IL-5, and INF- γ gene (Glue et al., 2002; Braun et al., 2013; Hoppin et al., 2013; North et al., 2014). DEHP and BBP have been shown to interfere with immunity against infection and alter the response of T helper type 2 (Th2) to increase allergic responses by acting on human plasmacytoid DCs (pDCs) by suppressing IFN-α/IFN-β expression and regulating the ability to elicit T-cell responses (Kuo et al., 2013).

#### Cancer

Phthalates have been implicated in the development of several types of cancer because of their xenoestrogenic properties-breast cancer in women and liver, skin, and gastrointestinal cancers in general population (Ardies and Dees, 1998; Lopez-Carrillo et al., 2010). A study that included 233 women residing in northern Mexico found exposure to DEP (the parent compound of MEP) was associated with increased risk of breast cancer with phthalate metabolites detected in at least 82% of the women (Lopez-Carrillo et al., 2010). MEP urinary concentrations were positively associated with breast cancer [odds ratio (OR), highest vs. lowest tertile = 2.20; 95% confidence interval (CI), 1.33–3.63; *p* for trend < 0.01] (Lopez-Carrillo et al., 2010).

Phthalates damage DNA in animal and human mammary epithelial cells which causes genomic instability in the breast tissue (Konduracka et al., 2014). Phthalates act as agonists for PPARs and activate the BARC gene through molecular signaling (Guyton et al., 2009; Rusyn and Corton, 2012; Sarath Josh et al., 2014). DEHP at high doses (100 and 500 μM) impaired the efficacy of camptothecin (CPT), an antitumor agent and reduced CPT- induced formation of reactive oxygen species (ROS) in ERα-positive MCF-7 cells (Chou et al., 2019). The impaired response of CPT in DEHP- exposed MCF-7 cells was mediated by epigenetic changes. MCF-7 cells after 48 hrs of exposure to 100 μM DEHP displayed considerable changes in patterns of DNA methylation, including hypermethylation of 700 genes and hypomethylation of 221 genes (Chou et al., 2019). In a Danish nationwide cohort of 1.12 million women who were followed for 10 years, 84% of breast cancers were ER-positive, and high level DBP exposure (≥10,000 mg) was directly related with a 2-fold increase in the rate of estrogen receptor- positive breast cancer risk (Ahern et al., 2019). This *in vivo* observation is consistent with *in vitro* evidences of DBP induced increases in proliferation and viability in an ER-dependent MCF-7 breast cancer cell line (Hong et al., 2005; van Meeuwen et al., 2007; Chen and Chien, 2014; Chen et al., 2016).

## Phthalates and microRNAs

MicroRNAs (miRNAs) are single-stranded, non-coding RNA molecules (sncRNAs) which are evolutionary-conserved and are involved in the regulation of gene expression at the posttranscriptional level (Ambros, 2004; Macfarlane and Murphy, 2010). miRNAs are ~22 nucleotides long and can base-pair with complementary sequences of the 3' untranslated region (UTR) of messenger RNAs (mRNA) and thereby cause repression of translation and/or degradation of mRNA (Bird, 2007; Goldberg et al., 2007; Berger et al., 2009; Zhang and Ho, 2011).

One hypothesis is that the long-term reproductive defects associated with phthalate exposure is exerted through the action of non-coding miRNAs (Scarano et al., 2019). In that study, pregnant rats were dosed with phthalate mixture in the following proportion: 21% DEHP, 35% DEP, 15% DBP, 8% DiBP, 5% BBzP, and 15% DiNP. This proportion of phthalate mixture was based on proportion of phthalates metabolites detected in urine samples from pregnant women (Zhou C. et al., 2017; Scarano et al., 2019). To examine whether exposure to the phthalate mixture is capable of altering gene expression during prostate development of the filial generation, levels of mRNAs and miRNAs genome-wide were analyzed by RNA-seq (Scarano et al., 2019). The period of treatment was from gestational day 10 (DG10) to postnatal day 21 (DPN21) as development of urogenital tract especially the prostate occurs during this period (Vilamaior et al., 2006; Prins and Putz, 2008; Zhou C. et al., 2017; Scarano et al., 2019). Results indicated that the phthalate mixture induced changes in phenotypic parameters such as the AGD on PND1 and PND22 and prostate weight and testosterone levels at PND22 (Scarano et al., 2019). miR-184 was upregulated in all treated groups as opposed to control and miR-141-3p was upregulated only at the lowest dose. RNA sequencing analyses indicated that 120 genes were downregulated at the lowest dose with several of these genes associated with development, differentiation, and oncogenesis. A considerable number of the downregulated genes were predicted to be targets of miR-141-3p and miR-184, and the genes were induced at the lower exposure doses (Scarano et al., 2019). It was concluded that differentially expressed genes (DEG)s were under negative regulation either by the miRNAs which are upregulated or other mechanisms causing gene suppression (Scarano et al., 2019).

### Gestational Diabetes

Several circulating miRNAs are dysregulated in patients diagnosed with gestational diabetes mellitus (GDM) during pregnancy (Zhao et al., 2011; Zhu et al., 2015). A study sought to identify the association of BPA and phthalate exposure measured in serum with the expression of circulating miRNAs related to GDM (miR-9-5p, miR-16-5p, miR-29a-3p, and miR-330-3p) revealed higher levels of miR-9-5p, miR-29a-3p, and miR-330-3p of patients with GDM compared to non-diabetic subjects (Martinez-Ibarra et al., 2019). Phthalate metabolites like MBP, mono-isobutyl phthalate (MiBP), mono-benzyl phthalate (MBzP), and MEHP were detected in 97–100% of urine samples and Bisphenol-A (BPA) in only 40% of samples (Martinez-Ibarra et al., 2019). Thus, phthalates and BPA may play a role in the development of metabolic diseases like GDM via epigenetic regulatory mechanism such as miRNA regulation.

### Female Fertility

A cross-sectional study was carried out to assess whether biomarkers of phenols and phthalates in urine of women undergoing IVF treatment are correlated with expression of extracellular vesicles (EV)-miRNAs in their follicular fluid. The urine samples were collected from participants during ovarian stimulation and the day oocyte was retrieved (Martinez et al., 2019). Results indicated that hsa-miR-125b and hsa-miR-15b were positively related with DEHP, while levels of hsa-miR-106b, and hsa-miR-374a were inversely related with DEHP. MBP was positively associated with levels of hsa-miR-24. *hsa-let-7c* was positively associated with urinary concentrations of mono-2-ethyl-5-oxohexyl phthalate (MEOHP), mono-2-ethyl-5-hydroxyhexyl phthalate (MEHHP), mono-2-ethyl-5-carboxypentyl phthalate (MECPP), and DEHP (Martinez et al., 2019). EV-miRNAs are associated in cellular communication (both intra- and inter-) within the ovarian follicle and thus their dysregulation after EDC exposure can impact follicular growth, ovarian function and thus fertility rates.

### microRNAs and Placenta

Changes in mRNA levels by phthalates have also been correlated with placental function. In a population study composed of 179 pregnant women–newborn dyads, an analysis was conducted to investigate the association between 8 phenol and 11 phthalate metabolites measured in first trimester urine and expression of 29 candidate miRNAs in placenta (LaRocca et al., 2016; Strakovsky and Schantz, 2018). Three miRNAs—miR-142-3p, miR15a-5p, and miR-185 were significantly associated with phenol or phthalate levels and potential mRNA targets of these microRNAs were linked with several biological pathways like regulation of protein serine/threonine kinase activity (LaRocca et al., 2016). Another small study comprising of 10 twin pregnancies suggested that several maternal urinary phthalate metabolites, including mono(carboxy-isononyl) phthalate (MCNP), MEHP, MEHHP, MECPP, mono-2-ethyl-5-oxohexyl phthalate (MEOHP), MBzP, mono(carboxy-isooctyl) phthalate (MCOP), mono-hydroxyisobutyl phthalate (MHiBP), and MiBP were positively correlated with placental long non-coding RNAs (lncRNAs) (Machtinger et al., 2018).

Placenta-derived EV-miRNAs are released by the placenta into the maternal circulation during pregnancy, and are responsible for regulating the endocrine environment to facilitate pregnancy and fetal growth (Mitchell et al., 2015). An exploratory study revealed that maternal exposure to phthalates and parabens can alter the profile of circulating EV-miRNAs (Zhong et al., 2019). miR-518e is highly expressed in women with elevated urinary levels of monobenzyl phthalate and methyl paraben. miR-373-3p had the lowest expression in women exposed to high levels of methyl paraben while miR-543 showed significant downregulation in women with high levels of paraben metabolites (Zhong et al., 2019). miR-518e, a member of the C19MC family is restricted to the placenta and the reproductive system and has high expression levels in the placentas of women having preeclampsia (Yang et al., 2015; Vashukova et al., 2016).

## Biomonitoring and Comparative Toxicogenomics Database (CTD)

### Biomonitoring Phthalate Levels in Humans

Single spot urine samples comprising of excreted urinary metabolites contribute most of the data collected for biomonitoring of human phthalate exposure (McKee et al., 2004; Hauser and Calafat, 2005; Wormuth et al., 2006; Frederiksen et al., 2007). Short-branched phthalates are mainly excreted as its monoester phthalates via urine (Frederiksen et al., 2007). The long-branched phthalates undergo further hydroxylation and oxidation and are excreted in urine and feces as phase II conjugated compounds. The phase II conjugates can be catalyzed by the enzyme uridine 5'-diphosphoglucuronyl transferase to form the hydrophilic glucuronide conjugate and is excreted in urine (Silva et al., 2003; Koch et al., 2005). A single urine sample for measurement of phthalate metabolites is also not an accurate estimation for an individual's long-term exposure level (Meeker et al., 2009). There have been scarce reports so far about phthalate levels in fetal cord blood and amniotic fluid. Also, cord blood or placenta may not correctly represent fetal exposure during the vulnerable period (Latini et al., 2003b). Phthalates diesters and their metabolites have been measured in breast milk, cord blood, and other pregnancy related specimens in humans (Adibi et al., 2003; Latini et al., 2003b; Main et al., 2006). Amniotic fluid is primarily formed from fetal urination and metabolized fetal cells. The phthalate metabolite concentrations in amniotic fluid varies based on metabolic activities of both mother and fetus and placental transfer but none of these metabolic parameters have been so far characterized for phthalate metabolites (Huang et al., 2009). Routine amniocentesis is usually performed at 16–20 weeks of gestation and amniotic fluid obtained during that time may provide accurate fetal exposure assessment during a period of reproductive differentiation and organogenesis (Silva et al., 2004). In the same study, among 10 phthalate metabolites analyzed-mEP, mBP, and mEHP were detected in 18.5% of the amniotic fluid samples taken from 54 anonymous donors (Silva et al., 2004). Infact, mEP, mBP, and mEHP were also major phthalate metabolites detected in serum samples from a multiethnic population (Silva et al., 2003). In one Italian study of 84 newborns, it was observed that MEHP in the cord blood of the newborns was associated with shorter gestations (Latini et al., 2003b).

### The Comparative Toxicogenomics Database (CTD)

The Center for the Evaluation of Risks to Human Reproduction (CERHR) was established by the National Toxicology Program (NTP) in 1998 (National Toxicology Program, 2019). CERHR provides a public resource for information regarding adverse health effects caused by exposure to various environmental and occupational chemicals. The chemicals are nominated for evaluation based on extent of public concern, production volume, potential of human exposure from environmental sources and availability of database on reproductive and developmental toxicity studies of the chemical (National Toxicology Program, 2019). CERHR selected DEHP based on the fact that general population of the United States is exposed to DEHP levels ranging from 1 to 30 μg/kg bw/day and it has been estimated that infants are exposed to DEHP through medical procedures and exposure can be as high as 6,000 μg/kg bw/day (National Toxicology Program, 2019). The Phthalates Expert Panel completed the first CERHR panel evaluation of DEHP in 2000. CEHR selected DEHP because of widespread public and government interest in its adverse health outcomes and availability of several toxicity papers at that time (Singh and Li, 2011; National Toxicology Program, 2019).

The Comparative Toxicogenomics Database (CTD) is a curated database that aids in understanding the effects of various environmental chemicals on human health. Biocurators at CTD use information from the literature to manually curate chemical-gene interactions, chemical-disease relationships and gene-disease relationships and construct chemical-gene-disease networks (Davis et al., 2009). Biocurators at CTD maintain toxicogenomic data and curation focuses on environmental chemicals. It is composed of data collected from 270 species with over 116,000 interactions between 3,900 chemicals and 13,300 genes/proteins, 5,900 gene/protein– disease direct relationships, and 2,500 chemical–disease direct relationships (Singh and Li, 2011).

In the CTD database, five most frequently curated phthalates (DEHP/MEHP and DBP/BBP/MBP) along with BPA have 1,232 and 265 interactions with unique genes/proteins, respectively (Singh and Li, 2011, 2012a,b). In one study (Singh and Li, 2011), in order to understand the health impact of the five most abundantly found phthalates, the authors downloaded the curated interactions between the five most common phthalates and the genes /proteins from CTD. From this database, 249 phthalate-interacting genes/proteins were fully analyzed for their Gene Ontology (GO) pathways, networks, and human diseases inferred by the phthalate–gene/protein–disease relationships. This analysis has ascertained that the pathways and networks of the top 34 genes were very similar to those of the 249 unique genes. Thus, the top 34 genes may be regarded as molecular biomarkers of phthalate toxicity (Singh and Li, 2011). The developmental effects of DBP/BBP/MBP depend primarily on two different factors- the duration of exposure and age of the embryo at the time of exposure. In an attempt to study the embryo-toxicant MBP, ESCs were exposed from the early embryoid body stage to 24 h post exposure and RNA was collected after 6, 12, and 24 h of exposure to study gene expression profile (van Dartel et al., 2009). There were a total number of 43 genes that were upregulated in the study and those were functionally related to cardiomyocyte differentiation (van Dartel et al., 2009).

### No-Observable-Effect-Level (NOEL)/No-Observable-Adverse-Effect-Level (NOAEL)/Lowest-Observed-Adverse-Effect Level (LOAEL)

Regulatory agencies like U.S. Environmental Protection Agency (EPA), the National Toxicology Program (NTP) typically include three doses while testing chemicals such as environmental endocrine disruptors (EEDs) like phthalates for the purposes of non-clinical risk assessment: (i) No-Observable-Adverse-Effect-Level (NOAEL): the highest dose/exposure given to an organism found by experiment or observation that has no observed toxic or adverse effect on traditional toxicological endpoints compared to an appropriate control (ii) No-Observable-Effect-Level (NOEL): the highest dose or exposure level that produces no observable effect in the animals tested when compared with its appropriate control (iii) Lowest-Observed-Adverse-Effect Level (LOAEL): the lowest concentration of a substance that causes toxic or biochemical effects in animal studies (Vandenberg et al., 2012). The term “adverse effect” designates any harmful anatomical, biochemical, or functional changes caused in test subjects due to administration of the particular chemical used in that study (Kerlin et al., 2016).

Traditionally, NOAELs, NOELs, and LOAELs are calculated by first determining the maximum tolerated dose of a chemical, and then adjusting the dose downward until no adverse effects are observed (Vandenberg et al., 2012). This approach fails to identify non-monotonic dose responses that may be found in lower doses of endocrine disrupting chemicals like phthalates. A review of low-dose effects and non-monotonic dose curves of endocrine disrupting chemicals is available (Vandenberg et al., 2012). The endocrine system has evolved to respond to very low concentrations of unbound physiologically active hormones (Welshons et al., 2003). Natural hormones can affect their targets with serum levels in the nano and picomolar range. Likewise, EDCs often exert effects at doses in the nano to micromolar range, resulting in non-monotonic dose-responses that fail to align with predictions from higher doses (Vandenberg et al., 2012).

A dose response curve is termed non-monotonic when the slope of the curve changes sign one or more times within the range of doses examined (Vandenberg et al., 2012). Non-monotonic dose-response curves (NMDRCs) are either U-shaped, indicating that maximum responses of the measured endpoint are observed at low and high doses, or inverted U-shaped, indicating maximal responses are observed at intermediate doses (Vandenberg et al., 2012). NMDRCs are generated by a variety of mechanisms—(i) hormones though toxic at high doses can affect biological endpoints at very low doses (ii) two or more monotonic responses can overlap affecting a common endpoint in opposite directions via different pathways (iii) differences in receptor affinity at low *vs*. high doses (iv) downregulation of receptor and receptor desensitization (e.g., decrease in response to a hormone occurs due to biochemical inactivation of a receptor) (v) receptor competition, in that the mixture of endogenous hormones and EDCs creates an environment that gives rise to NMDRCs (Vandenberg et al., 2012).

The epigenome varies by cell, tissue and stage of development, and EDCs may impact different cells and tissues differently, and findings that identify differential methylation resulting from phthalate exposure may be confounded by cell composition of the biological sample (Breton et al., 2017). Further complicating the identification of NOAELs of phthalates in human epigenetic studies is the fact that humans are chronically exposed to low doses as opposed to the acute high-dose exposures used in animal studies (CDC, 2019). This makes the traditional NOAEL calculations challenging to apply to human studies. Two critical areas of research for identifying NOAELs of phthalates in human epigenetic research are the identification of EDC induced changes in the epigenome across varying tissues and the characterization of the mechanisms through which phthalates impact the epigenome. There are several factors that determine the success of high-throughput epigenome-wide association scans (EWAS) like Illumina Infinium HumanMethylation450 (Illumina 450K) e.g., sample size, statistical power, epigenetic risk effect size and differentially methylated regions (DMRs) (Tsai and Bell, 2015).

The effects of acute doses of phthalates often fail to manifest in the dosed generation but are noted in one to two generations after the original generation is dosed, and multigenerational animal studies are generally designed to evaluate such effects. Low-powered studies and low number of widely spaced dose groups can give rise to inaccurate NOAEL values (Barnes et al., 1995; Sand et al., 2002; Hotchkiss et al., 2008). One study (Blystone et al., 2010) designed to evaluate the effect of DEHP on male reproductive malformations (RTM)s in male Sprague-Dawley rats used more than three traditional dose groups plus control and a bigger than usual sample size of F1 and F2 male rats until adulthood to define the DEHP NOAEL for male RTMs and also to evaluate the shape of dose-response curve. The *in utero* exposures for F1 and F2 were the same and the NOAEL for F1 and F2 RTM combined data were 100 ppm (4.8 mg/kg/day), and the lowest observed adverse effect level (LOAEL) was 300 ppm or 14 mg/kg/day (Blystone et al., 2010).

Furthermore, NOAELs vary according to the different endpoint parameters studied (Zhang et al., 2004). A particular study designed to evaluate the effect of DBP on reproductive and developmental toxicity during gestational day (GD1) to postnatal day (PND21) on F1 male rats showed that the NOAEL was 250 mg/kg/day when endpoint measured was number of live pups per litter whereas the NOAEL was 50 mg/kg/day when endpoint measured was birth weight of live pups (Zhang et al., 2004). These multigenerational animal models demonstrate that the calculation of NOAELs requires specific endpoints to delineate adverse effect. DNA methylation can be viewed as a process that results from the exposure and contributes to the eventual expression of an endpoint but is not the endpoint itself. As DNA methylation is not an endpoint in health research but is an intermediary between phthalate exposure to other endpoints such as gene-expression, determination of NOAELs for epigenetic research will need to reflect the degrees to which changes in methylation adversely affect these end-points, which are yet unknown. Perhaps a more important question to answer is do we need to establish NOEALs for epigenetic research if the changes in DNA methylation are not the ultimate outcomes we seek to understand.

## Discussion

This review is expected to inspire future research endeavors in environmental epigenetics to investigate the effects of the endocrine disruptors at different life stages from the perspective of transgenerational and multigenerational epigenetic inheritance (Table 1). There is substantial evidence from ESTs and rodent models that phthalates disrupt healthy growth and development of a fetus. There is more limited human evidence, though there is a body of literature that demonstrates associations between phthalate exposure and disrupted growth and development. Experimental evidence also demonstrates that the effects of phthalate exposure may not be realized until later in the life-cycle or in subsequent generations. Experimental and observational data demonstrate that exposure to phthalates modifies gene expression through epigenetic changes such as DNA methylation at CpG sites. One of the main areas of concern is maternal exposure of phthalates to fetus and infants via placenta and breast milk (Latini et al., 2003a; Calafat et al., 2006).

**Table 1:** Research studies of the epigenetic impact of those phthalates and the long-term health consequences of exposure to phthalates by model.

**Model**	**Life-cycle timing of impact of phthalate exposure**	**Impact of exposure**	**Epigenetic dysregulation associated with exposure**	**Associated phthalate di-esters or monoesters**	**Analysis method**	**References**
**Embryo and embryonic stem cell models**						
Embryonic stem cells (Murine)	Embryonic stage	Inhibition of mesoderm-derived cardiomyocyte differentiation	Upregulated gene expression of 43 genes	MBP	Microarray analysis & Gene Set Enrichment Analysis (GSEA)	van Dartel et al., 2009
Embryonic stem cells (Human)	Embryonic stage	Cytotoxic and affected the development of hESCs	Changed gene expression patterns in embryoid bodies (EB)	MEHP	Gene expression patterns analyzed by real-time PCR	Shi et al., 2013
Embryo (Murine)	Embryonic stage	Impaired developmental competency, delayed progression of preimplantation, increase in reactive oxygen species, increased apoptosis	Decreased DNA methylation	MBP	Immunofluorescent staining& quantification of immunofluorescent intensity	Chu et al., 2013
**Placenta models**						
Placenta (human)	Fetal stage	Placental function	Altered methylation and gene expression in human placenta	Total urinary phthalate concentration	Illumina Infinium HM 850k BeadChip	Grindler et al., 2018
Placenta, Cord blood	Fetal stage	No association with fetal length or birthweight	Decreased methylation *H19* in women with high levels of total urinary phthalate concentrations Total phthalates and low molecular weight phthalates associated with decreased methylation of *IGF2*DMR0	11 phthalate metabolites(MBzP, MEHP, MEHHP, MECPP, MEOHP, MnBP, MiBP, MBzP, MEP, MCOP, MCPP, MCNP).	Methylation of differentially methylated regions (DMRs) were assessed by pyrosequencing of *H19, IGF2*DMR0, and *IGF2*DMR2	LaRocca et al., 2014
Placenta (Human)	Fetal & neonatal stages	Fetal growth restriction (FGR) newborns	Inverse association of urinary phthalate concentrations with *IGF2* DNA methylation in human placenta	MEHHPMEOHP	PCR & pyrosequencing	Zhao et al., 2016
Placenta (Human)	Placental and fetal growth	Gene ontology (GO) identified biological pathways to health outcomes	Three miRNAs were significantly associated with phthalate levels (miR-185, miR-142-3p, miR15a-5p)	11 phthalate metabolites(MBzP,MEHP, MEHHP,MECPP, MEOHP, MnBP, MiBP, MBzP, MEP, MCOP, MCPP, MCNP).	qPCR	LaRocca et al., 2016
Placenta (Human)	Newborn stage	long non-coding RNAs (lncRNA)s play an important role in regulating genomic imprinting	lncRNAs	MCNP,MEHP, MECPP, MEOHP, MBzP, MCOP, MHiBP, MiBP, MMP, MCPP,MEP, MNP, MnBP, MHBP	Real-time PCR	Machtinger et al., 2018
**Blood-based models**						
Peripheral Blood Mononuclear Cells (human), monocytic cell line THP-1	Adult birch-pollen allergic and non-allergic individuals	Increased inflammatory cytokine gene expression	A significant increase in IL-4, IL-5 and INF- γ gene expression were observed	MBEP, MBUP, MEHP, MOP, MINP, MIDP	Quantitative competitive RT-PCR and real-time PCR	Glue et al., 2002
Whole blood from umbilical cord at birth and children at 9 years	Fetal stage	Asthma, inflammation, restricted child growth, and poor sperm quality	Inverse association between MEP concentration and cord blood Alu repeats Inverse association between DEHP and Alu repeat methylation in children at 9 years of age	MEP, MBP, MiBP, MEHP, MEHHP, MEOHP, MECPP, MBzP, MCPP, MCOP, MCNP	Pyrosequencing	Huen et al., 2016
Whole blood from umbilical cord	Fetal stage	Genes related to androgen response, estrogen response, spermatogenesis enriched	Altered DNA methylation	DEHP	HM450K	Chen et al., 2018
Whole blood from children	Childhood	Decreased methylation of TNF-α gene promoter and childhood asthma	Detection of DNA methylation by pyrosequencing, real-time PCR	MEHP	Quantitative PCR	Wang et al., 2015
Whole blood from children	Childhood	Skinfold thickness in girls 8 to 14 years old.No direct link between phthalate exposures and adiposity measures mediated by changes in DNA methylation	Altered DNA methylation of H19 in girls	MEP,MBP, MiBP, MCPP, MBzP, MEHP, MEHHP,MEOHP, MECPP	Pyrosequencing	Bowman et al., 2019
Serum from pregnant women	During gestation	Higher levels of miR-9-5p, miR-29a-3p and miR-330-3p in sera of patients with gestational diabetes mellitus compared to non-diabetic subjects	miRNA expression	MBP, MiBP, MBzP, MEHP	Real-Time PCR	Martinez-Ibarra et al., 2019
**Murine pregnancy models**						
Pregnant rats	F1 generation reproductive stage	Adult testicular function	Hypermethylation in SF-1 and Sp-1 promoter regions of Leydig cells	DEHP	Real-Time PCR	Sekaran and Jagadeesan, 2015
Pregnant rats	F1 generation reproductive stage	Adult male testicular and prostate disease	Altered DNA methylation in sperm and transgenerational inheritance	DEHP	Quantitative PCR	Manikkam et al., 2013)
Pregnant rats	F1 generation reproductive stage	Genes controlling immune response affected by *in utero* DEHP exposure	DNA methylation alterations throughout epigenome of adult male adrenal glands	DEHP	Reduced-representation bisulfite sequencing	Martinez-Arguelles and Papadopoulos, 2015
Pregnant mice	F2 generation childhood	Allergic airway inflammation	Altered DNA methylation and transgenerational model	BBP	MassARRAY	Jahreis et al., 2018
Pregnant rats	F1 generation	Low and high exposure groups had higher body weight than control group	Altered DNA methylation of Srebf1 and Srebf2	DEP,DEHP, DBP, DiNP, DiBP, BBP	EZ DNA Methylation Gold Kit	Moody et al., 2019
Pregnant rats	F1 Male reproductive stage	Altered ano-genital distance, prostate weight, and testosterone levels	Non-coding miRNA	Mixture of DEHP,DEP, DBP, DiBP, BBzP, DiNP	(i) RNAs sequenced by HiSeq2500 platform (Illumina) (ii) High performance sequencing—sncRNAs (NovaSeq Sequencing System)	Scarano et al., 2019
**Other models**						
Follicular fluid	Female reproductive stage	Dysregulation of follicular growth, ovarian function, and fertility	EV-miRNA	DEHP,MBP, MEOHP,MEHHP, MECPP	TaqMan Open Array Human microRNA panel	Martinez-Ibarra et al., 2019
Spermatozoa	Reproductive stage	Genes associated with growth and development, and basic cellular function, and diminished blastocyst quality	Differential DNA methylation	MEHP, MEOHP, MBP, MCOCH	HM450K	Wu et al., 2017a
Placental derived extracellular vehicles circulating in maternal blood	Fetal stage	Expression of mi-518e associated with increased BBP	EV-miRNA	BBP	TaqMan Open Array Human microRNA panel	Zhong et al., 2019
Mouse liver and testes	Adult reproductive stage	DEHP causes toxicity in liver- liver is involved in steroid metabolism and is known to be a DEHP target organ.	51 DEHP-regulated genes were identified involved in-peroxisome proliferation, xenobiotic detoxification, oxidative stress response, immune function, steroid hormone metabolism, testis development, and pheromone transport	DEHP	Analysis of DEHP induced gene expression changes in liver using microarray screening of Murine Genome U74Av2 Arrays (MGU74Av2) (Affymetrix, Santa Clara, CA)	Wong and Gill, 2002

*hESC, Human embryonic stem cell; EB, Embryoid Body; EV, Extracellular Vesicle*.

Environmental exposure to phthalates causes developmental and reproductive toxicity in rodent studies, though such relationships are difficult to demonstrate in humans. Life in all mammals occurs in cycles: production of germ cells (sperm and eggs) followed by fertilization, gestational development of the embryo, birth, postnatal growth followed by puberty leading to sexual maturity and the ability to reproduce. Though the terms “developmental” and “reproductive” toxicities are two separate entities, there is substantial overlap between them (Shelby, 2006). Toxicologists in recent years prefer to conduct their studies in a life-cycle specific manner because it is a common occurrence that chemical exposure at one stage of the life cycle may lead to observable effects at a later stage (Akingbemi et al., 2001). The adverse effect of phthalates on the early development of male reproductive tract affecting expression of genes involved in testis development and steroid hormone synthesis has been of particular interest (Wong and Gill, 2002; Shelby, 2006; Sekaran and Jagadeesan, 2015). Any chemical-induced epigenetic defects in eggs or sperm in a sexually mature individual might not affect the individual but may be transmitted to its progenies. That effect of the chemical might be embryonic lethality, miscarriage, stillbirth or the offspring might be born with a developmental disorder.

Most of the evidences we currently have for transgenerational epigenetic inheritance is in animals. In humans, because of our long lifespan and diverse genetics, it is complicated to conduct studies for 3 to 4 generations (Calo et al., 1993). From this perspective, the zebrafish provides an ideal model as it has a short time to sexual maturity (~3–4 months). Moreover, zebrafish eggs get fertilized externally in water and thus are at exposed environment and so F0 fish is equivalent to F1 mice. This enables us to examine the effect of the exposed environmental toxin directly at F0 (De Felice et al., 2002).

Transgenerational studies with other EDCs like dioxin or TCDD which is a persistent environmental toxicant show that unexposed TCDD-lineage F2 offspring have defects in reproduction, skeletal muscle system and sex ratio in offspring. Moreover, the decrease in fertility and egg release in control female zebrafish is due to the unexposed, TCDD-lineage F2 male zebrafish. The ancestral TCDD exposure affects reproductive success of male zebrafish across multiple generations (De Felice et al., 2002). In a follow up study, the transgenerational effect of TCDD on zebrafish reproductive success was found to be the result of altered DNA methylation (De Felice et al., 1999). The authors performed whole genome methylation analysis of adult zebrafish exposed to sublethal levels of TCDD during the developmental period and found both DMR- and CpG-specific changes in the DNA methylation profile. The authors observed that several genes were differentially methylated in the exposed compared to the unexposed, and many of those genes were responsible for reproductive success or epigenetic modifications (De Felice et al., 1999). Thus, similar transgenerational studies are required for phthalates.

The epigenetic tags of the chromatin are of two types—(i) transient which can be removed and (ii) permanent which is heritable (Ayala-Garcia et al., 2013). The transient epigenetic tags enable the organism to adjust their gene expression status in relation to changes in their environment—in contrast permanent epigenetic tags gives rise to an epigenetic memory which modulates the cells' genetic and metabolic response to environmental changes for the rest of the organism's life (Ayala-Garcia et al., 2013). When permanent chromatin epigenetic tags occur in the stem cells, gametes—they are inherited by their progenies both at the cellular and organismal levels and are thus transgenerationally heritable (Dolinoy et al., 2007; McCarrey, 2012). Thus, the highly dynamic process of epigenetic tagging as perpetuated by the epigenetic memory is responsible for the phenotypic plasticity in an organism. Tagging occurs as a response to changes in environmental conditions at any time point in the life course (Ayala-Garcia et al., 2013). Humans are simultaneously exposed to several xenobiotics and thus the interaction of phthalates with other environmental toxins should be taken into account when studying their roles in causing diseases in the population (Benjamin et al., 2017). One major drawback is that epidemiological studies are mostly limited to developed countries (Benjamin et al., 2017). We have limited reports so far on the impact of phthalates from Asia, Africa and South America. In a 2018 study of Children's Health and Environmental Chemicals in Korea (CHECK) cohort, comprising of matched pregnant woman-fetus pairs recruited from four cities of Korea, phthalate metabolites like MiBP, MnBP, MEHP, MEHHP, and MEOHP (metabolites of DEHP), MEP, persistent organic pollutants (POPs), heavy metals, and BPA were linked with decreased neurodevelopmental performances and behavioral scores of toddlers (Kim et al., 2018). In another Mothers and Children's Environmental Health Study composed of 460 mother–infant pairs between 2006 and 2009 revealed that prenatal exposure to phthalates is inversely associated with the Mental and Psychomotor Developmental indices (MDI and PDI, respectively) of particularly male infants, at 6 months as measured by the Korean Bayley Scales of Infant Development (Bayley, 1993; Park, 2006; Kim et al., 2018).

The European Union (EU) has adopted strict regulations around use of phthalates and other EDCs- it is an interesting natural experiment that could be used to investigate how these diseases change in Europe. The impact of phthalates varies from one population to population based on their food habits and lifestyles. The concentration of phthalate metabolites in human body widely varies based on demographics (Benjamin et al., 2017). A joint, co-ordinated and conscious effort by various nations is needed to manage the existing health issues caused by EDCs like phthalates across the globe plus the onset of new cases which will result in huge expenditures for a nation's economy in the years to come. A Steering Committee of scientists in the EU evaluated a range of health and economic costs due to EDC exposures based on epidemiological and toxicological evidences. The disease and dysfunctionality of life caused due to EDC exposures in the EU is estimated to cost hundreds of billions of Euros per year (Trasande et al., 2015). As a global population, we should adapt the 5 R's (Reduce, Reuse, Recycle, Rethink, and Restrain) for controlling the environmental exposure to phthalates and other EDCs for our future generations.

## Future Perspectives

An emerging view in the field of epidemiologic research is that intrauterine growth period of the fetus is a critical window of susceptibility during which environmental toxicants can affect the developmental trajectories and cause epigenetic information to be transmitted between generations (Morkve Knudsen et al., 2018). Germ cells undergo extensive epigenetic reprogramming starting from embryonic stage to mature reproductive stage and are vulnerable to environmental stressors during those reprogramming phases (Wu et al., 2015). A true transgenerational event is one in which epigenetic information is transmitted across generations through the germline, and is known to occur when a man or woman (F0) and their germ cells to the F1 generation are directly subjected to any environmental stressor and the F2 offspring is the first generation which is a true case for transgenerational epigenetic inheritance (Horsthemke, 2018; Morkve Knudsen et al., 2018).

The various mechanisms which play an important role in passing on information from one generation to another are DNA methylation, histone modification, or changes in non-coding RNA (Heard and Martienssen, 2014; Wu et al., 2015; Sales et al., 2017; Horsthemke, 2018). Intergenerational effects occur when a pregnant woman (F0) is subjected to environmental stress, and the developing fetus including the germline of the fetus may be affected leading to altered phenotype of the child (F1) and possibly the next generation (F2). Intergenerational epigenetic inheritance is the transfer of epigenetic marks from the gametes to the embryo for only one generation. The third generation (F3) is the first generation that could exhibit transgenerational epigenetic inheritance (Morkve Knudsen et al., 2018). Multigenerational exposures are exposure related events observed across multiple generations (Skinner, 2008).

Where can research improve to develop a better understanding of the biological mechanisms underpinning phthalate exposures and human disease? Environmental epidemiology needs to focus on accurate characterization of phthalate exposure by using multiple samples across time to best capture exposure status. Epigenetic studies of phthalate exposures should consider how mixtures of phthalates affect gene expression beyond the traditional classifications of low and high molecular weight.

In conclusion, future studies of phthalates and other environmental chemicals must examine potential multigenerational effects of exposures. Maternal exposures, both prior to and during pregnancy, can potentially affect the developing egg and fetus. Paternal exposures can potentially affect the sperm. More studies with model organisms, such as with zebrafish, are needed to examine the mechanisms of multigenerational inheritance of phenotypes induced by chemicals such as phthalates.

## Author Contributions

SD conducted the literature review, drafted, and revised the manuscript. DH provided critical review of the manuscript and revised the manuscript. DAR provided critical review of the manuscript. DMR conceived the review, provided critical review of the manuscript, and revised the manuscript.

## Conflict of Interest

DAR is the Chief Scientific Officer in the Reproductive Stress company, but the collaboration on this project involves no conflict of interest. The remaining authors also declare that the research was conducted in the absence of any commercial or financial relationships that could be construed as a potential conflict of interest.

## Glossary of Acronyms

Phthalates
BBP: Benzyl butyl phthalate
BBzP: Butylbenzyl-phthalate
BzBP: Benzylbutyl phthalate
DBP: Dibutyl phthalate
DCHP: Dicyclohexyl phthalate
DEHP: Di-(2-ethylhexyl) phthalate
DEP: Diethyl phthalate
DiBP: Diisobutyl phthalate
DiDP: Di-isodecyl phthalate
DiNP: Di-isononyl phthalate
DMP: Dimethyl phthalate
DnOP: Di-n-octyl phthalate
mBP-glu: Monobutyl phthalate glucuronide
mBzP: Mono-benzyl phthalate
mBzP-glu: Monobenzyl phthalate glucuronide
MCNP: Mono (carboxy-isononyl) phthalate
MCOP: Mono (carboxy-isooctyl) phthalate
MECPP: Mono-2-ethyl-5-carboxypentyl phthalate
MEHHP: Mono-2-ethyl-5-hydroxyhexyl phthalate
MEHP: Mono-2-Ethylhexyl Phthalate
MEOHP: Mono (2-ethyl-5-oxohexyl) phthalate
MEP: Mono ethyl phthalate
MHiBP: Mono-hydroxyisobutyl phthalate
MiBP: Mono-isobutyl phthalate
MiDP: Mono-iso-decyl phthalate
MiNP: Mono-isononyl phthalate
MnBP: Mono-n-butyl phthalate
MOP: Mono-n-octyl phthalate
Others
ADAMTS: aA Disintegrin and Metalloproteinase with Thrombospondin Motifs
AGD: Anogenital distance
BPA: Bisphenol A
5caC: 5-carboxylcytosine
CERHR: Center for the Evaluation of Risks to Human Reproduction
CTD: Comparative Toxicogenomics Database
DC: Dendritic cells
DMR: Differentially methylated regions
DNMTs: DNA methyltransferases
EDCs: Endocrine disrupting chemicals
ESCs: Embryonic stem cells
E_2_: Estrogen
ER: Estrogen receptor
5fC: 5-formylcytosine
GDM: Gestational diabetes mellitus
HM450K: Human Methylation 450K BeadChip
HMW: High molecular weight
huESCs: Human embryonic stem cells
5hmC: 5-hydroxymethylcytosine
IGF2: Insulin-like growth-factor 2
IL: Interleukin
LMW: Low molecular weight
LINE-1: Long interspersed nucleotide elements
5mC: 5-methylcytosine
miRNAs: microRNA
NTP: National Toxicology Program
PBMC: Peripheral blood mononuclear cells
PVC: Polyvinyl chloride
P_4_: Progesterone
SREBPs: Sterol regulatory element binding proteins
TET: Ten-eleven translocation
TNF-α: Tumor necrosis factor-α

## References

[B1] Adibi J. J. Perera F. P. Jedrychowski W. Camann D. E. Barr D. Jacek R.. (2003). Prenatal exposures to phthalates among women in New York City and Krakow, Poland. Environ. Health Perspect. 111, 1719–1722. 10.1289/ehp.6235 14594621 PMC1241713

[B2] Ahern T. P. Broe A. Lash T. L. Cronin-Fenton D. P. Ulrichsen S. P. Christiansen P. M.. (2019). Phthalate exposure and breast cancer incidence: a Danish Nationwide Cohort study. J. Clin. Oncol. 37, 1800–1809. 10.1200/JCO.18.02202 30995175 PMC7351345

[B3] Akingbemi B. T. Youker R. T. Sottas C. M. Ge R. Katz E. Klinefelter G. R.. (2001). Modulation of rat Leydig cell steroidogenic function by di(2-ethylhexyl)phthalate. Biol. Reprod. 65, 1252–1259. 10.1095/biolreprod65.4.1252 11566751

[B4] Ambros V. (2004). The functions of animal microRNAs. Nature 431, 350–355. 10.1038/nature02871 15372042

[B5] Anderson A. M. Carter K. W. Anderson D. Wise M. J. (2012). Coexpression of nuclear receptors and histone methylation modifying genes in the testis: implications for endocrine disruptor modes of action. PLoS ONE 7:e34158. 10.1371/journal.pone.0034158 22496781 PMC3319570

[B6] Andrade A. J. Grande S. W. Talsness C. E. Grote K. Chahoud I. (2006). A dose-response study following *in utero* and lactational exposure to di-(2-ethylhexyl)-phthalate (DEHP): non-monotonic dose-response and low dose effects on rat brain aromatase activity. Toxicology 227, 185–192. 10.1016/j.tox.2006.07.022 16949715

[B7] Araki A. Mitsui T. Miyashita C. Nakajima T. Naito H. Ito S.. (2014). Association between maternal exposure to di(2-ethylhexyl) phthalate and reproductive hormone levels in fetal blood: the Hokkaido study on environment and children's health. PLoS ONE 9:e109039. 10.1371/journal.pone.0109039 25296284 PMC4189794

[B8] Ardies C. M. Dees C. (1998). Xenoestrogens significantly enhance risk for breast cancer during growth and adolescence. Med. Hypotheses 50, 457–464. 10.1016/S0306-9877(98)90262-6 9710315

[B9] ATSDR (1995). Toxicological Profile for diethyl phthalate (DEP). Atlanta, GA. Available online at: http://www.atsdr.cdc.gov/toxprofiles

[B10] ATSDR (2001). Toxicological Profile for di-n-butyl phthalate (DBP). Atlanta, GA. Available online at: http://www.atsdr.cdc.gov/toxprofiles 37931045

[B11] ATSDR (2002). Toxicological Profile for di(2-ethylhexyl)phthalate (DEHP). Atlanta, GA. Available online at: http://www.atsdr.cdc.gov/toxprofiles 37040457

[B12] Ayala-Garcia B. Lopez-Santibanez Guevara M. Marcos-Camacho L. I. Fuentes-Farias A. L. Melendez-Herrera E. Gutierrez-Ospina G. (2013). Speciation, phenotypic variation and plasticity: what can endocrine disruptors tell us? Int. J. Endocrinol. 2013:862739. 10.1155/2013/862739 23762055 PMC3670528

[B13] Baccarelli A. Ghosh S. (2012). Environmental exposures, epigenetics and cardiovascular disease. Curr. Opin. Clin. Nutr. Metab. Care 15, 323–329. 10.1097/MCO.0b013e328354bf5c 22669047 PMC3742092

[B14] Barker D. J. (1997). Maternal nutrition, fetal nutrition, and disease in later life. Nutrition 13, 807–813. 10.1016/S0899-9007(97)00193-7 9290095

[B15] Barnes D. G. Daston G. P. Evans J. S. Jarabek A. M. Kavlock R. J. Kimmel C. A.. (1995). Benchmark Dose Workshop: criteria for use of a benchmark dose to estimate a reference dose. Regul. Toxicol. Pharmacol. 21, 296–306. 10.1006/rtph.1995.1043 7644719

[B16] Bayley N. (1993). Bayley Scales of Infant Development, 2nd Edn. San Antonio, TX: Psychological Corporation.

[B17] Benjamin S. Masai E. Kamimura N. Takahashi K. Anderson R. C. Faisal P. A. (2017). Phthalates impact human health: Epidemiological evidences and plausible mechanism of action. J. Haz. Mater. 340, 360–383. 10.1016/j.jhazmat.2017.06.036 28800814

[B18] Berger S. L. Kouzarides T. Shiekhattar R. Shilatifard A. (2009). An operational definition of epigenetics. Genes Dev. 23, 781–783. 10.1101/gad.1787609 19339683 PMC3959995

[B19] Berry M. A. Hargadon B. Shelley M. Parker D. Shaw D. E. Green R. H.. (2006). Evidence of a role of tumor necrosis factor alpha in refractory asthma. N. Engl. J. Med. 354, 697–708. 10.1056/NEJMoa050580 16481637

[B20] Bestor T. Laudano A. Mattaliano R. Ingram V. (1988). Cloning and sequencing of a cDNA encoding DNA methyltransferase of mouse cells. The carboxyl-terminal domain of the mammalian enzymes is related to bacterial restriction methyltransferases. J. Mol. Biol. 203, 971–983. 10.1016/0022-2836(88)90122-2 3210246

[B21] Biemann R. Fischer B. Navarrete Santos A. (2014). Adipogenic effects of a combination of the endocrine-disrupting compounds bisphenol A, diethylhexylphthalate, and tributyltin. Obes. Facts 7, 48–56. 10.1159/000358913 24503497 PMC5644809

[B22] Bird A. (2007). Perceptions of epigenetics. Nature 447, 396–398. 10.1038/nature05913 17522671

[B23] Blumberg B. (2011). Obesogens, stem cells and the maternal programming of obesity. J. Dev. Orig. Health Dis. 2, 3–8. 10.1017/S2040174410000589 26401242 PMC4576931

[B24] Blystone C. R. Kissling G. E. Bishop J. B. Chapin R. E. Wolfe G. W. Foster P. M. (2010). Determination of the di-(2-ethylhexyl) phthalate NOAEL for reproductive development in the rat: importance of the retention of extra animals to adulthood. Toxicol. Sci. 116, 640–646. 10.1093/toxsci/kfq147 20484383 PMC2905405

[B25] Bouman A. Heineman M. J. Faas M. M. (2005). Sex hormones and the immune response in humans. Hum. Reprod. Update 11, 411–423. 10.1093/humupd/dmi008 15817524

[B26] Bowman A. Peterson K. E. Dolinoy D. C. Meeker J. D. Sanchez B. N. Mercado-Garcia A.. (2019). Phthalate exposures, DNA methylation and adiposity in mexican children through adolescence. Front. Public Health 7:162. 10.3389/fpubh.2019.00162 31275917 PMC6593088

[B27] Braun J. M. Sathyanarayana S. Hauser R. (2013). Phthalate exposure and children's health. Curr. Opin. Pediatr. 25, 247–254. 10.1097/MOP.0b013e32835e1eb6 23429708 PMC3747651

[B28] Breton C. V. Marsit C. J. Faustman E. Nadeau K. Goodrich J. M. Dolinoy D. C.. (2017). Small-magnitude effect sizes in epigenetic end points are important in children's environmental health studies: The Children's Environmental Health and Disease Prevention Research Center's Epigenetics Working Group. Environ. Health Perspect. 125, 511–526. 10.1289/EHP595 28362264 PMC5382002

[B29] Broide D. H. Lotz M. Cuomo A. J. Coburn D. A. Federman E. C. Wasserman S. I. (1992). Cytokines in symptomatic asthma airways. J. Allergy Clin. Immunol. 89, 958–967. 10.1016/0091-6749(92)90218-Q 1374772

[B30] Buckley J. P. Engel S. M. Braun J. M. Whyatt R. M. Daniels J. L. Mendez M. A.. (2016). Prenatal phthalate exposures and body mass index among 4- to 7-year-old children: a pooled analysis. Epidemiology 27, 449–458. 10.1097/EDE.0000000000000436 26745610 PMC4821741

[B31] Buckley J. P. Palmieri R. T. Matuszewski J. M. Herring A. H. Baird D. D. Hartmann K. E.. (2012). Consumer product exposures associated with urinary phthalate levels in pregnant women. J. Exp. Sci. Environ. Epidemiol. 22, 468–475. 10.1038/jes.2012.33 22760436 PMC3439834

[B32] Cai H. Zheng W. Zheng P. Wang S. Tan H. He G.. (2015). Human urinary/seminal phthalates or their metabolite levels and semen quality: a meta-analysis. Environ. Res. 142, 486–494. 10.1016/j.envres.2015.07.008 26275958

[B33] Calafat A. M. Brock J. W. Silva M. J. Gray L. E. Jr. Reidy J. A. Barr D. B.. (2006). Urinary and amniotic fluid levels of phthalate monoesters in rats after the oral administration of di(2-ethylhexyl) phthalate and di-n-butyl phthalate. Toxicology 217, 22–30. 10.1016/j.tox.2005.08.013 16171919

[B34] Calo L. Fracasso A. Cantaro S. Cozzi E. De Silvestro G. Plebani M.. (1993). Plasticizers induced mononuclear cells interleukin 1 production: implications with peritoneal sclerosis. Clin. Nephrol. 40:57. 8358875

[B35] Cantonwine D. E. Meeker J. D. Ferguson K. K. Mukherjee B. Hauser R. McElrath T. F. (2016). Urinary concentrations of bisphenol A and phthalate metabolites measured during pregnancy and risk of preeclampsia. Environ. Health Perspect. 124, 1651–1655. 10.1289/EHP188 27177253 PMC5047771

[B36] Carreras E. Turner S. Paharkova-Vatchkova V. Mao A. Dascher C. Kovats S. (2008). Estradiol acts directly on bone marrow myeloid progenitors to differentially regulate GM-CSF or Flt3 ligand-mediated dendritic cell differentiation. J. Immunol. 180, 727–738. 10.4049/jimmunol.180.2.727 18178810

[B37] Carruthers C. M. Foster P. M. (2005). Critical window of male reproductive tract development in rats following gestational exposure to di-n-butyl phthalate. Birth Defects Res. B Dev. Reprod. Toxicol. 74, 277–285. 10.1002/bdrb.20050 15954088

[B38] CDC (2019). Fourth Report on Human Exposures to Environmental Chemicals, Updated Tables, Centers for Disease Control and Prevention. Atlanta, GA.

[B39] Chapin R. E. Robbins W. A. Schieve L. A. Sweeney A. M. Tabacova S. A. Tomashek K. M. (2004). Off to a good start: the influence of pre- and periconceptional exposures, parental fertility, and nutrition on children's health. Environ. Health Perspect. 112, 69–78. 10.1289/ehp.6261 14698934 PMC1241800

[B40] Chen C. H. Jiang S. S. Chang I. S. Wen H. J. Sun C. W. Wang S. L. (2018). Association between fetal exposure to phthalate endocrine disruptor and genome-wide DNA methylation at birth. Environ. Res. 162, 261–270. 10.1016/j.envres.2018.01.009 29367177

[B41] Chen F. P. Chien M. H. (2014). Lower concentrations of phthalates induce proliferation in human breast cancer cells. Climacteric 17, 377–384. 10.3109/13697137.2013.865720 24228746

[B42] Chen F. P. Chien M. H. Chern I. Y. (2016). Impact of low concentrations of phthalates on the effects of 17beta-estradiol in MCF-7 breast cancer cells. Taiwan. J. Obstet. Gynecol. 55, 826–834. 10.1016/j.tjog.2015.11.003 28040128

[B43] Chen J. A. Liu H. Qiu Z. Shu W. (2008). Analysis of di-n-butyl phthalate and other organic pollutants in Chongqing women undergoing parturition. Environ. Pollut. 156, 849–853. 10.1016/j.envpol.2008.05.019 18565632

[B44] Chen Y. Xue F. (2018). The impact of gestational hypothyroxinemia on the cognitive and motor development of offspring. J. Matern. Fetal Neonatal Med. 33, 1940–1945. 10.1080/14767058.2018.1529749 30348031

[B45] Chou C. K. Huang H. W. Yang C. F. Dahms H. U. Liang S. S. Wang T. N.. (2019). Reduced camptothecin sensitivity of estrogen receptor-positive human breast cancer cells following exposure to di(2-ethylhexyl)phthalate (DEHP) is associated with DNA methylation changes. Environ. Toxicol. 34, 401–414. 10.1002/tox.22694 30720231

[B46] Chu D. P. Tian S. Sun D. G. Hao C. J. Xia H. F. Ma X. (2013). Exposure to mono-n-butyl phthalate disrupts the development of preimplantation embryos. Reprod. Fertil. Dev. 25, 1174–1184. 10.1071/RD12178 23231764

[B47] Cimmino L. Abdel-Wahab O. Levine R. L. Aifantis I. (2011). TET family proteins and their role in stem cell differentiation and transformation. Cell Stem Cell 9, 193–204. 10.1016/j.stem.2011.08.007 21885017 PMC3244690

[B48] Cirillo T. Fasano E. Esposito F. Montuori P. Amodio Cocchieri R. (2013). Di(2-ethylhexyl)phthalate (DEHP) and di-n-butylphthalate (DBP) exposure through diet in hospital patients. Food Chem. Toxicol. 51, 434–438. 10.1016/j.fct.2012.10.015 23108212

[B49] Cobellis L. Latini G. De Felice C. Razzi S. Paris I. Ruggieri F.. (2003). High plasma concentrations of di-(2-ethylhexyl)-phthalate in women with endometriosis. Hum. Reprod. 18, 1512–1515. 10.1093/humrep/deg254 12832380

[B50] Das S. Bonaguidi M. Muro K. Kessler J. A. (2008). Generation of embryonic stem cells: limitations of and alternatives to inner cell mass harvest. Neurosurg. Focus 24:E4. 10.3171/FOC/2008/24/3-4/E3 18341407

[B51] Davis A. P. Murphy C. G. Saraceni-Richards C. A. Rosenstein M. C. Wiegers T. C. Mattingly C. J. (2009). Comparative Toxicogenomics Database: a knowledgebase and discovery tool for chemical-gene-disease networks. Nucleic Acids Res. 37, D786–D792. 10.1093/nar/gkn580 18782832 PMC2686584

[B52] De Felice C. Latini G. Toti P. D'Addario V. Petraglia F. Bagnoli F. (2002). Small thymus at birth and gestational age. Eur. J. Pediatr. 161, 362–363. 10.1007/s00431-002-0948-2 12029463

[B53] De Felice C. Toti P. Santopietro R. Stumpo M. Pecciarini L. Bagnoli F. (1999). Small thymus in very low birth weight infants born to mothers with subclinical chorioamnionitis. J. Pediatr. 135, 384–386. 10.1016/S0022-3476(99)70140-X 10484809

[B54] Desvergne B. Feige J. N. Casals-Casas C. (2009). PPAR-mediated activity of phthalates: a link to the obesity epidemic? Mol. Cell. Endocrinol. 304, 43–48. 10.1016/j.mce.2009.02.017 19433246

[B55] Dewalque L. Charlier C. Pirard C. (2014). Estimated daily intake and cumulative risk assessment of phthalate diesters in a Belgian general population. Toxicol. Lett. 231, 161–168. 10.1016/j.toxlet.2014.06.028 24968065

[B56] Di Ciaula A. Portincasa P. (2019). Diet and contaminants: driving the rise to obesity epidemics? Curr. Med. Chem. 26, 3471–3482. 10.2174/0929867324666170518095736 28521687

[B57] Dolinoy D. C. Huang D. Jirtle R. L. (2007). Maternal nutrient supplementation counteracts bisphenol A-induced DNA hypomethylation in early development. Proc. Natl. Acad. Sci. U.S.A. 104, 13056–13061. 10.1073/pnas.0703739104 17670942 PMC1941790

[B58] Doyle T. J. Bowman J. L. Windell V. L. McLean D. J. Kim K. H. (2013). Transgenerational effects of di-(2-ethylhexyl) phthalate on testicular germ cell associations and spermatogonial stem cells in mice. Biol. Reprod. 88:112. 10.1095/biolreprod.112.106104 23536373 PMC4013901

[B59] Du Y. Y. Fang Y. L. Wang Y. X. Zeng Q. Guo N. Zhao H.. (2016). Follicular fluid and urinary concentrations of phthalate metabolites among infertile women and associations with *in vitro* fertilization parameters. Reprod. Toxicol. 61, 142–150. 10.1016/j.reprotox.2016.04.005 27067915

[B60] Ema M. Miyawaki E. Hirose A. Kamata E. (2003). Decreased anogenital distance and increased incidence of undescended testes in fetuses of rats given monobenzyl phthalate, a major metabolite of butyl benzyl phthalate. Reprod. Toxicol. 17, 407–412. 10.1016/S0890-6238(03)00037-6 12849851

[B61] Engel S. M. Miodovnik A. Canfield R. L. Zhu C. Silva M. J. Calafat A. M.. (2010). Prenatal phthalate exposure is associated with childhood behavior and executive functioning. Environ. Health Perspect. 118, 565–571. 10.1289/ehp.0901470 20106747 PMC2854736

[B62] Factor-Litvak P. Insel B. Calafat A. M. Liu X. Perera F. Rauh V. A.. (2014). Persistent associations between maternal prenatal exposure to phthalates on child IQ at age 7 years. PLoS ONE 9:e114003. 10.1371/journal.pone.0114003 25493564 PMC4262205

[B63] Ferguson K. K. McElrath T. F. Meeker J. D. (2014). Environmental phthalate exposure and preterm birth. JAMA Pediatr. 168, 61–67. 10.1001/jamapediatrics.2013.3699 24247736 PMC4005250

[B64] Food U. S. Drug Administration (2001). Safety Assessment of Di(2-ethylhexyl)phthalate (DEHP) Released from PVC Medical Devices. Rockville, MD: Food U. S. and Drug Administration. Available online at: http://www.fda.gov/Safety/MedWatch/SafetyInformation/SafetyAlertsforHumanMedicalProducts/ucm154574.htm (accessed September 5, 2001).

[B65] Frederiksen H. Skakkebaek N. E. Andersson A. M. (2007). Metabolism of phthalates in humans. Mol. Nutr. Food Res. 51, 899–911. 10.1002/mnfr.200600243 17604388

[B66] Frederiksen H. Sorensen K. Mouritsen A. Aksglaede L. Hagen C. P. Petersen J. H.. (2012). High urinary phthalate concentration associated with delayed pubarche in girls. Int. J. Androl. 35, 216–226. 10.1111/j.1365-2605.2012.01260.x 22428786

[B67] Gesta S. Bluher M. Yamamoto Y. Norris A. W. Berndt J. Kralisch S.. (2006). Evidence for a role of developmental genes in the origin of obesity and body fat distribution. Proc. Natl. Acad. Sci. U.S.A. 103, 6676–6681. 10.1073/pnas.0601752103 16617105 PMC1458940

[B68] Ghassabian A. Trasande L. (2018). Disruption in thyroid signaling pathway: a mechanism for the effect of endocrine-disrupting chemicals on child neurodevelopment. Front. Endocrinol. 9:204. 10.3389/fendo.2018.00204 29760680 PMC5936967

[B69] Gillman M. W. Barker D. Bier D. Cagampang F. Challis J. Fall C.. (2007). Meeting report on the 3rd International Congress on Developmental Origins of Health and Disease (DOHaD). Pediatr. Res. 61, 625–629. 10.1203/pdr.0b013e3180459fcd 17413866

[B70] Gluckman P. D. Hanson M. A. (2004). Living with the past: evolution, development, and patterns of disease. Science 305, 1733–1736. 10.1126/science.1095292 15375258

[B71] Glue C. Millner A. Bodtger U. Jinquan T. Poulsen L. K. (2002). *In vitro* effects of monophthalates on cytokine expression in the monocytic cell line THP-1 and in peripheral blood mononuclear cells from allergic and non-allergic donors. Toxicol. In Vitro 16, 657–662. 10.1016/S0887-2333(02)00082-6 12423647

[B72] Goldberg A. D. Allis C. D. Bernstein E. (2007). Epigenetics: a landscape takes shape. Cell 128, 635–638. 10.1016/j.cell.2007.02.006 17320500

[B73] Goncalves L. F. Chaiworapongsa T. Romero R. (2002). Intrauterine infection and prematurity. Ment. Retard. Dev. Disabil. Res. Rev. 8, 3–13. 10.1002/mrdd.10008 11921380

[B74] Gore A. C. Chappell V. A. Fenton S. E. Flaws J. A. Nadal A. Prins G. S.. (2015). Executive summary to EDC-2: the endocrine society's second scientific statement on endocrine-disrupting chemicals. Endocr. Rev. 36, 593–602. 10.1210/er.2015-1093 26414233 PMC4702495

[B75] Gray L. E. Jr. Ostby J. Furr J. Price M. Veeramachaneni D. N. Parks L. (2000). Perinatal exposure to the phthalates DEHP, BBP, and DINP, but not DEP, DMP, or DOTP, alters sexual differentiation of the male rat. Toxicol. Sci. 58, 350–365. 10.1093/toxsci/58.2.350 11099647

[B76] Grindler N. M. Vanderlinden L. Karthikraj R. Kannan K. Teal S. Polotsky A. J.. (2018). Exposure to phthalate, an endocrine disrupting chemical, alters the first trimester placental methylome and transcriptome in women. Sci. Rep. 8:6086. 10.1038/s41598-018-24505-w 29666409 PMC5904105

[B77] Grygiel-Gorniak B. (2014). Peroxisome proliferator-activated receptors and their ligands: nutritional and clinical implications–a review. Nutr. J. 13:17. 10.1186/1475-2891-13-17 24524207 PMC3943808

[B78] Gu H. Liu Y. Wang W. Ding L. Teng W. Liu L. (2016). *In utero* exposure to di-(2-ethylhexyl) phthalate induces metabolic disorder and increases fat accumulation in visceral depots of C57BL/6J mice offspring. Exp. Ther. Med. 12, 3806–3812. 10.3892/etm.2016.3820 28105114 PMC5228422

[B79] Gutierrez-Garcia A. K. Flores-Kelly J. M. Ortiz-Rodriguez T. Kalixto-Sanchez M. A. De Leon-Rodriguez A. (2019). Phthalates affect the *in vitro* expansion of human hematopoietic stem cell. Cytotechnology 71, 553–561. 10.1007/s10616-019-00300-x 30715687 PMC6465380

[B80] Guyton K. Z. Chiu W. A. Bateson T. F. Jinot J. Scott C. S. Brown R. C.. (2009). A reexamination of the PPAR-alpha activation mode of action as a basis for assessing human cancer risks of environmental contaminants. Environ. Health Perspect. 117, 1664–1672. 10.1289/ehp.0900758 20049115 PMC2801168

[B81] Hauser R. Calafat A. M. (2005). Phthalates and human health. Occup. Environ. Med. 62, 806–818. 10.1136/oem.2004.017590 16234408 PMC1740925

[B82] Hauser R. Gaskins A. J. Souter I. Smith K. W. Dodge L. E. Ehrlich S.. (2016). Urinary phthalate metabolite concentrations and reproductive outcomes among women undergoing *in vitro* fertilization: results from the EARTH study. Environ. Health Perspect. 124, 831–839. 10.1289/ehp.1509760 26545148 PMC4892919

[B83] Heard E. Martienssen R. A. (2014). Transgenerational epigenetic inheritance: myths and mechanisms. Cell 157, 95–109. 10.1016/j.cell.2014.02.045 24679529 PMC4020004

[B84] Hillman L. S. Goodwin S. L. Sherman W. R. (1975). Identification and measurement of plasticizer in neonatal tissues after umbilical catheters and blood products. N. Engl. J. Med. 292, 381–386. 10.1056/NEJM197502202920801 1110722

[B85] Hon G. C. Song C. X. Du T. Jin F. Selvaraj S. Lee A. Y.. (2014). 5mC oxidation by Tet2 modulates enhancer activity and timing of transcriptome reprogramming during differentiation. Mol. Cell 56, 286–297. 10.1016/j.molcel.2014.08.026 25263596 PMC4319980

[B86] Hong E. J. Ji Y. K. Choi K. C. Manabe N. Jeung E. B. (2005). Conflict of estrogenic activity by various phthalates between *in vitro* and *in vivo* models related to the expression of Calbindin-D9k. J. Reprod. Dev. 51, 253–263. 10.1262/jrd.16075 15883486

[B87] Hong S. H. Lee J. E. Kim H. S. Jung Y. J. Hwang D. Lee J. H.. (2016). Effect of vitamin D3 on production of progesterone in porcine granulosa cells by regulation of steroidogenic enzymes. J. Biomed. Res. 30, 203–208. 10.7555/JBR.30.2016K0012 27533930 PMC4885168

[B88] Hoppin J. A. Jaramillo R. London S. J. Bertelsen R. J. Salo P. M. Sandler D. P.. (2013). Phthalate exposure and allergy in the U.S. population: results from NHANES 2005-2006. Environ. Health Perspect. 121, 1129–1134. 10.1289/ehp.1206211 23799650 PMC3801456

[B89] Horsthemke B. (2018). A critical view on transgenerational epigenetic inheritance in humans. Nat. Commun. 9:2973. 10.1038/s41467-018-05445-5 30061690 PMC6065375

[B90] Hotchkiss A. K. Rider C. V. Blystone C. R. Wilson V. S. Hartig P. C. Ankley G. T.. (2008). Fifteen years after “Wingspread”–environmental endocrine disrupters and human and wildlife health: where we are today and where we need to go. Toxicol. Sci. 105, 235–259. 10.1093/toxsci/kfn030 18281716 PMC2721670

[B91] Huang P. C. Kuo P. L. Chou Y. Y. Lin S. J. Lee C. C. (2009). Association between prenatal exposure to phthalates and the health of newborns. Environ. Int. 35, 14–20. 10.1016/j.envint.2008.05.012 18640725

[B92] Huang P. C. Li W. F. Liao P. C. Sun C. W. Tsai E. M. Wang S. L. (2014). Risk for estrogen-dependent diseases in relation to phthalate exposure and polymorphisms of CYP17A1 and estrogen receptor genes. Environ. Sci. Pollut. Res. Int. 21, 13964–13973. 10.1007/s11356-014-3260-6 25030786

[B93] Huang Y. Chavez L. Chang X. Wang X. Pastor W. A. Kang J.. (2014). Distinct roles of the methylcytosine oxidases Tet1 and Tet2 in mouse embryonic stem cells. Proc. Natl. Acad. Sci. U.S.A. 111, 1361–1366. 10.1073/pnas.1322921111 24474761 PMC3910590

[B94] Huen K. Calafat A. M. Bradman A. Yousefi P. Eskenazi B. Holland N. (2016). Maternal phthalate exposure during pregnancy is associated with DNA methylation of LINE-1 and Alu repetitive elements in Mexican-American children. Environ. Res. 148, 55–62. 10.1016/j.envres.2016.03.025 27019040 PMC4874877

[B95] Iqbal K. Tran D. A. Li A. X. Warden C. Bai A. Y. Singh P.. (2015). Deleterious effects of endocrine disruptors are corrected in the mammalian germline by epigenome reprogramming. Genome Biol. 16:59. 10.1186/s13059-015-0619-z 25853433 PMC4376074

[B96] Ito S. D'Alessio A. C. Taranova O. V. Hong K. Sowers L. C. Zhang Y. (2010). Role of Tet proteins in 5mC to 5hmC conversion, ES-cell self-renewal and inner cell mass specification. Nature 466, 1129–1133. 10.1038/nature09303 20639862 PMC3491567

[B97] Ito T. Inoue K. Nishimura N. Takano H. (2012). Phthalate esters modulate the differentiation and maturation of mouse peripheral blood mononuclear cell-derived dendritic cells. J. Appl. Toxicol. 32, 142–148. 10.1002/jat.1652 21538406

[B98] Jaenisch R. Hochedlinger K. Blelloch R. Yamada Y. Baldwin K. Eggan K. (2004). Nuclear cloning, epigenetic reprogramming, and cellular differentiation. Cold Spring Harb. Symp. Quant. Biol. 69, 19–27. 10.1101/sqb.2004.69.19 16117629

[B99] Jahreis S. Trump S. Bauer M. Bauer T. Thurmann L. Feltens R.. (2018). Maternal phthalate exposure promotes allergic airway inflammation over 2 generations through epigenetic modifications. J. Allergy Clin. Immunol. 141, 741–753. 10.1016/j.jaci.2017.03.017 28392331

[B100] Janesick A. Blumberg B. (2011). Minireview: PPARgamma as the target of obesogens. J. Steroid Biochem. Mol. Biol. 127, 4–8. 10.1016/j.jsbmb.2011.01.005 21251979 PMC3116997

[B101] Janesick A. Blumberg B. (2012). Obesogens, stem cells and the developmental programming of obesity. Int. J. Androl. 35, 437–448. 10.1111/j.1365-2605.2012.01247.x 22372658 PMC3358413

[B102] Jeon S. Y. Hwang K. A. Kim C. W. Jeung E. B. Choi K. C. (2017). Altered expression of epithelial mesenchymal transition and pluripotent associated markers by sex steroid hormones in human embryonic stem cells. Mol. Med. Rep. 16, 828–836. 10.3892/mmr.2017.6672 28586020

[B103] Johnson K. J. McDowell E. N. Viereck M. P. Xia J. Q. (2011). Species-specific dibutyl phthalate fetal testis endocrine disruption correlates with inhibition of SREBP2-dependent gene expression pathways. Toxicol. Sci. 120, 460–474. 10.1093/toxsci/kfr020 21266533 PMC3061485

[B104] Kato K. Silva M. J. Reidy J. A. Hurtz D. III. Malek N. A. Needham L. L.. (2004). Mono(2-ethyl-5-hydroxyhexyl) phthalate and mono-(2-ethyl-5-oxohexyl) phthalate as biomarkers for human exposure assessment to di-(2-ethylhexyl) phthalate. Environ. Health Perspect. 112, 327–330. 10.1289/ehp.6663 14998748 PMC1241862

[B105] Kerlin R. Bolon B. Burkhardt J. Francke S. Greaves P. Meador V.. (2016). Scientific and regulatory policy committee: recommended (“best”) practices for determining, communicating, and using adverse effect data from nonclinical studies. Toxicol. Pathol. 44, 147–162. 10.1177/0192623315623265 26704930

[B106] Kim J. H. Park H. Lee J. Cho G. Choi S. Choi G.. (2016). Association of diethylhexyl phthalate with obesity-related markers and body mass change from birth to 3 months of age. J Epidemiol Community Health 70, 466–472. 10.1136/jech-2015-206315 26834143 PMC4862064

[B107] Kim S. Eom S. Kim H. J. Lee J. J. Choi G. Choi S.. (2018). Association between maternal exposure to major phthalates, heavy metals, and persistent organic pollutants, and the neurodevelopmental performances of their children at 1 to 2years of age- CHECK cohort study. Sci. Total Environ. 624, 377–384. 10.1016/j.scitotenv.2017.12.058 29258038

[B108] Kim Y. Ha E. H. Kim E. J. Park H. Ha M. Kim J. H.. (2011). Prenatal exposure to phthalates and infant development at 6 months: prospective Mothers and Children's Environmental Health (MOCEH) study. Environ. Health Perspect. 119, 1495–1500. 10.1289/ehp.1003178 21737372 PMC3230435

[B109] Koch H. M. Bolt H. M. Preuss R. Angerer J. (2005). New metabolites of di(2-ethylhexyl)phthalate (DEHP) in human urine and serum after single oral doses of deuterium-labelled DEHP. Arch. Toxicol. 79, 367–376. 10.1007/s00204-004-0642-4 15700144

[B110] Koch H. M. Lorber M. Christensen K. L. Palmke C. Koslitz S. Bruning T. (2013). Identifying sources of phthalate exposure with human biomonitoring: results of a 48h fasting study with urine collection and personal activity patterns. Int. J. Hyg. Environ. Health 216, 672–681. 10.1016/j.ijheh.2012.12.002 23333758

[B111] Konduracka E. Krzemieniecki K. Gajos G. (2014). Relationship between everyday use cosmetics and female breast cancer. Pol. Arch. Med. Wewn. 124, 264–269. 10.20452/pamw.2257 24694726

[B112] Kriaucionis S. Heintz N. (2009). The nuclear DNA base 5-hydroxymethylcytosine is present in Purkinje neurons and the brain. Science 324, 929–930. 10.1126/science.1169786 19372393 PMC3263819

[B113] Kuo C. H. Hsieh C. C. Kuo H. F. Huang M. Y. Yang S. N. Chen L. C.. (2013). Phthalates suppress type I interferon in human plasmacytoid dendritic cells via epigenetic regulation. Allergy 68, 870–879. 10.1111/all.12162 23738920

[B114] LaRocca J. Binder A. M. McElrath T. F. Michels K. B. (2014). The impact of first trimester phthalate and phenol exposure on IGF2/H19 genomic imprinting and birth outcomes. Environ. Res. 133, 396–406. 10.1016/j.envres.2014.04.032 24972507 PMC4155603

[B115] LaRocca J. Binder A. M. McElrath T. F. Michels K. B. (2016). First-trimester urine concentrations of phthalate metabolites and phenols and placenta miRNA expression in a cohort of U.S. Women. Environ. Health Perspect. 124, 380–387. 10.1289/ehp.1408409 26090578 PMC4786977

[B116] Latini G. (2000). Potential hazards of exposure to di-(2-ethylhexyl)-phthalate in babies. a review. Biol. Neonate 78, 269–276. 10.1159/000014278 11093005

[B117] Latini G. Avery G. B. (1999). Materials degradation in endotracheal tubes: a potential contributor to bronchopulmonary dysplasia. Acta Paediatr 88, 1174–1175. 10.1111/j.1651-2227.1999.tb01011.x 10565474

[B118] Latini G. De Felice C. Presta G. Del Vecchio A. Paris I. Ruggieri F.. (2003a). Exposure to Di(2-ethylhexyl)phthalate in humans during pregnancy. A preliminary report. Biol. Neonate 83, 22–24. 10.1159/000067012 12566679

[B119] Latini G. De Felice C. Presta G. Del Vecchio A. Paris I. Ruggieri F.. (2003b). *In utero* exposure to di-(2-ethylhexyl)phthalate and duration of human pregnancy. Environ. Health Perspect. 111, 1783–1785. 10.1289/ehp.6202 14594632 PMC1241724

[B120] Lee H. K. Kim T. S. Kim C. Y. Kang I. H. Kim M. G. Jung K. K.. (2012). Evaluation of *in vitro* screening system for estrogenicity: comparison of stably transfected human estrogen receptor-alpha transcriptional activation (OECD TG455) assay and estrogen receptor (ER) binding assay. J. Toxicol. Sci. 37, 431–437. 10.2131/jts.37.431 22467034

[B121] Levie D.T Korevaar I. M. Bath S. C. Dalmau-Bueno A. Murcia M. Guxens M.. (2018). Thyroid function in early pregnancy, child IQ, and autistic traits: a meta-analysis of individual participant data. J. Clin. Endocrinol. Metab. 103, 2967–2979. 10.1210/jc.2018-00224 29757392

[B122] Lin L. Zheng L. X. Gu Y. P. Wang J. Y. Zhang Y. H. Song W. M. (2008). [Levels of environmental endocrine disruptors in umbilical cord blood and maternal blood of low-birth-weight infants]. Zhonghua Yu Fang Yi Xue Za Zhi 42, 177–180. 18788582

[B123] Lio C. J. Rao A. (2019). TET enzymes and 5hmC in adaptive and innate immune systems. Front. Immunol. 10:210. 10.3389/fimmu.2019.00210 30809228 PMC6379312

[B124] Loff S. Kabs F. Witt K. Sartoris J. Mandl B. Niessen K. H.. (2000). Polyvinylchloride infusion lines expose infants to large amounts of toxic plasticizers. J. Pediatr. Surg. 35, 1775–1781. 10.1053/jpsu.2000.19249 11101735

[B125] Lopez-Carrillo L. Hernandez-Ramirez R. U. Calafat A. M. Torres-Sanchez L. Galvan-Portillo M. Needham L. L.. (2010). Exposure to phthalates and breast cancer risk in northern Mexico. Environ. Health Perspect. 118, 539–544. 10.1289/ehp.0901091 20368132 PMC2854732

[B126] Macfarlane L. A. Murphy P. R. (2010). MicroRNA: biogenesis, function and role in cancer. Curr. Genomics 11, 537–561. 10.2174/138920210793175895 21532838 PMC3048316

[B127] Machtinger R. Zhong J. Mansur A. Adir M. Racowsky C. Hauser R.. (2018). Placental lncRNA expression is associated with prenatal phthalate exposure. Toxicol. Sci. 163, 116–122. 10.1093/toxsci/kfy013 29385630 PMC5917778

[B128] Maggio M. Snyder P. J. Ceda G. P. Milaneschi Y. Luci M. Cattabiani C.. (2013). Is the haematopoietic effect of testosterone mediated by erythropoietin? The results of a clinical trial in older men. Andrology 1, 24–28. 10.1111/j.2047-2927.2012.00009.x 23258626

[B129] Main K. M. Mortensen G. K. Kaleva M. M. Boisen K. A. Damgaard I. N. Chellakooty M.. (2006). Human breast milk contamination with phthalates and alterations of endogenous reproductive hormones in infants three months of age. Environ. Health Perspect. 114, 270–276. 10.1289/ehp.8075 16451866 PMC1367843

[B130] Manikkam M. Tracey R. Guerrero-Bosagna C. Skinner M. K. (2013). Plastics derived endocrine disruptors (BPA, DEHP and DBP) induce epigenetic transgenerational inheritance of obesity, reproductive disease and sperm epimutations. PLoS ONE 8:e55387. 10.1371/journal.pone.0055387 23359474 PMC3554682

[B131] Maroziene L. Grazuleviciene R. (2002). Maternal exposure to low-level air pollution and pregnancy outcomes: a population-based study. Environ. Health 1:6. 10.1186/1476-069X-1-6 12495448 PMC149395

[B132] Marsee K. Woodruff T. J. Axelrad D. A. Calafat A. M. Swan S. H. (2006). Estimated daily phthalate exposures in a population of mothers of male infants exhibiting reduced anogenital distance. Environ. Health Perspect. 114, 805–809. 10.1289/ehp.8663 16759976 PMC1480516

[B133] Martinez R. M. Hauser R. Liang L. Mansur A. Adir M. Dioni L.. (2019). Urinary concentrations of phenols and phthalate metabolites reflect extracellular vesicle microRNA expression in follicular fluid. Environ. Int. 123, 20–28. 10.1016/j.envint.2018.11.043 30481674 PMC6343661

[B134] Martinez-Arguelles D. B. Culty M. Zirkin B. R. Papadopoulos V. (2009). *In utero* exposure to di-(2-ethylhexyl) phthalate decreases mineralocorticoid receptor expression in the adult testis. Endocrinology 150, 5575–5585. 10.1210/en.2009-0847 19819939 PMC2795714

[B135] Martinez-Arguelles D. B. Papadopoulos V. (2015). Identification of hot spots of DNA methylation in the adult male adrenal in response to *in utero* exposure to the ubiquitous endocrine disruptor plasticizer di-(2-ethylhexyl) phthalate. Endocrinology 156, 124–133. 10.1210/en.2014-1436 25330100

[B136] Martinez-Ibarra A. Martinez-Razo L. D. Vazquez-Martinez E. R. Martinez-Cruz N. Flores-Ramirez R. Garcia-Gomez E.. (2019). Unhealthy levels of phthalates and bisphenol A in Mexican pregnant women with gestational diabetes and its association to altered expression of miRNAs involved with metabolic disease. Int. J. Mol. Sci. 20, 1–17. 10.3390/ijms20133343 31284700 PMC6650872

[B137] Martino-Andrade A. J. Liu F. Sathyanarayana S. Barrett E. S. Redmon J. B. Nguyen R. H.. (2016). Timing of prenatal phthalate exposure in relation to genital endpoints in male newborns. Andrology 4, 585–593. 10.1111/andr.12180 27062102

[B138] Masuyama H. Hiramatsu Y. Kodama J. Kudo T. (2003). Expression and potential roles of pregnane X receptor in endometrial cancer. J. Clin. Endocrinol. Metab. 88, 4446–4454. 10.1210/jc.2003-030203 12970323

[B139] McCarrey J. R. (2012). The epigenome as a target for heritable environmental disruptions of cellular function. Mol. Cell. Endocrinol. 354, 9–15. 10.1016/j.mce.2011.09.014 21970811

[B140] McKee R. H. Butala J. H. David R. M. Gans G. (2004). NTP center for the evaluation of risks to human reproduction reports on phthalates: addressing the data gaps. Reprod. Toxicol. 18, 1–22. 10.1016/j.reprotox.2003.09.002 15013060

[B141] Meehan R. R. Thomson J. P. Lentini A. Nestor C. E. Pennings S. (2018). DNA methylation as a genomic marker of exposure to chemical and environmental agents. Curr. Opin. Chem. Biol. 45, 48–56. 10.1016/j.cbpa.2018.02.006 29505975

[B142] Meeker J. D. Sathyanarayana S. Swan S. H. (2009). Phthalates and other additives in plastics: human exposure and associated health outcomes. Philos. Trans. R. Soc. Lond. B. Biol. Sci. 364, 2097–2113. 10.1098/rstb.2008.0268 19528058 PMC2873014

[B143] Melekoglu R. Yilmaz E. Ciftci O. Kafadar Y. T. Celik E. (2019). Associations between second-trimester amniotic fluid levels of ADAMTS4, ADAMTS5, IL-6, and TNF-alpha and spontaneous preterm delivery in singleton pregnancies. J. Perinat. Med. 47, 304–310. 10.1515/jpm-2018-0297 30730845

[B144] Mierzejewska K. Borkowska S. Suszynska E. Suszynska M. Poniewierska-Baran A. Maj M.. (2015). Hematopoietic stem/progenitor cells express several functional sex hormone receptors-novel evidence for a potential developmental link between hematopoiesis and primordial germ cells. Stem Cells Dev. 24, 927–937. 10.1089/scd.2014.0546 25607657 PMC4390002

[B145] Mitchell M. D. Peiris H. N. Kobayashi M. Koh Y. Q. Duncombe G. Illanes S. E.. (2015). Placental exosomes in normal and complicated pregnancy. Am. J. Obstet. Gynecol. 213, S173–S181. 10.1016/j.ajog.2015.07.001 26428497

[B146] Moody L. Hernandez-Saavedra D. Kougias D. G. Chen H. Juraska J. M. Pan Y. X. (2019). Tissue-specific changes in Srebf1 and Srebf2 expression and DNA methylation with perinatal phthalate exposure. Environ. Epigenet. 5:dvz009. 10.1093/eep/dvz009 31240115 PMC6586200

[B147] Morkve Knudsen T. Rezwan F. I. Jiang Y. Karmaus W. Svanes C. Holloway J. W. (2018). Transgenerational and intergenerational epigenetic inheritance in allergic diseases. J. Allergy Clin. Immunol. 142, 765–772. 10.1016/j.jaci.2018.07.007 30040975 PMC6167012

[B148] Mu D. Gao F. Fan Z. Shen H. Peng H. Hu J. (2015). Levels of phthalate metabolites in urine of pregnant women and risk of clinical pregnancy loss. Environ. Sci. Technol. 49, 10651–10657. 10.1021/acs.est.5b02617 26251123

[B149] Mylchreest E. Cattley R. C. Foster P. M. (1998). Male reproductive tract malformations in rats following gestational and lactational exposure to Di(n-butyl) phthalate: an antiandrogenic mechanism? Toxicol. Sci. 43, 47–60. 10.1006/toxs.1998.2436 9629619

[B150] Nakada D. Oguro H. Levi B. P. Ryan N. Kitano A. Saitoh Y.. (2014). Oestrogen increases haematopoietic stem-cell self-renewal in females and during pregnancy. Nature 505, 555–558. 10.1038/nature12932 24451543 PMC4015622

[B151] National Toxicology Program (2019). NTP-CERHR Monograph on the Potential Human Reproductive and Developmental Effects of Di(2-ethylhexyl) Phthalate (DEHP). NIH Pub. No. 06–4476. 19407857

[B152] Nelissen E. C. van Montfoort A. P. Dumoulin J. C. Evers J. L. (2011). Epigenetics and the placenta. Hum. Reprod. Update 17, 397–417. 10.1093/humupd/dmq052 20959349

[B153] Nestor C. E. Ottaviano R. Reddington J. Sproul D. Reinhardt D. Dunican D.. (2012). Tissue type is a major modifier of the 5-hydroxymethylcytosine content of human genes. Genome Res. 22, 467–477. 10.1101/gr.126417.111 22106369 PMC3290782

[B154] Ng M. Fleming T. Robinson M. Thomson B. Graetz N. Margono C.. (2014). Global, regional, and national prevalence of overweight and obesity in children and adults during 1980-2013: a systematic analysis for the Global Burden of Disease Study 2013. Lancet 384, 766–781. 10.1016/S0140-6736(14)60460-8 24880830 PMC4624264

[B155] Nilsson E. Larsen G. Manikkam M. Guerrero-Bosagna C. Savenkova M. I. Skinner M. K. (2012). Environmentally induced epigenetic transgenerational inheritance of ovarian disease. PLoS ONE 7:e36129. 10.1371/journal.pone.0036129 22570695 PMC3343040

[B156] North M. L. Takaro T. K. Diamond M. L. Ellis A. K. (2014). Effects of phthalates on the development and expression of allergic disease and asthma. Ann. Allergy Asthma Immunol. 112, 496–502. 10.1016/j.anai.2014.03.013 24726194

[B157] Okano M. Bell D. W. Haber D. A. Li E. (1999). DNA methyltransferases Dnmt3a and Dnmt3b are essential for *de novo* methylation and mammalian development. Cell 99, 247–257. 10.1016/S0092-8674(00)81656-6 10555141

[B158] Park B. H. (2006). Korean Bayley Scales of Infant Development. Interpretation Manual, 2nd Edn. Seoul: KIDSPOP Publishing Corporation.

[B159] Pidsley R. Zotenko E. Peters T. J. Lawrence M. G. Risbridger G. P. Molloy P.. (2016). Critical evaluation of the Illumina MethylationEPIC BeadChip microarray for whole-genome DNA methylation profiling. Genome Biol. 17:208. 10.1186/s13059-016-1066-1 27717381 PMC5055731

[B160] Plonait S. L. Nau H. Maier R. F. Wittfoht W. Obladen M. (1993). Exposure of newborn infants to di-(2-ethylhexyl)-phthalate and 2-ethylhexanoic acid following exchange transfusion with polyvinylchloride catheters. Transfusion 33, 598–605. 10.1046/j.1537-2995.1993.33793325058.x 8333024

[B161] Prados J. Stenz L. Somm E. Stouder C. Dayer A. Paoloni-Giacobino A. (2015). Prenatal exposure to DEHP affects spermatogenesis and sperm DNA methylation in a strain-dependent manner. PLoS ONE 10:e0132136. 10.1371/journal.pone.0132136 26244509 PMC4526524

[B162] Prezioso G. Giannini C. Chiarelli F. (2018). Effect of thyroid hormones on neurons and neurodevelopment. Horm. Res. Paediatr. 90, 73–81. 10.1159/000492129 30157487

[B163] Prins G. S. Putz O. (2008). Molecular signaling pathways that regulate prostate gland development. Differentiation 76, 641–659. 10.1111/j.1432-0436.2008.00277.x 18462433 PMC2824174

[B164] Radford E. J. Ito M. Shi H. Corish J. A. Yamazawa K. Isganaitis E.. (2014). *In utero* effects. *In utero* undernourishment perturbs the adult sperm methylome and intergenerational metabolism. Science 345:1255903. 10.1126/science.1255903 25011554 PMC4404520

[B165] Rajesh P. Balasubramanian K. (2014). Phthalate exposure *in utero* causes epigenetic changes and impairs insulin signalling. J. Endocrinol. 223, 47–66. 10.1530/JOE-14-0111 25232145

[B166] Ronzoni S. Steckle V. D'Souza R. Murphy K. E. Lye S. Shynlova O. (2018). Cytokine changes in maternal peripheral blood correlate with time-to-delivery in pregnancies complicated by premature prelabor rupture of the membranes. Reprod. Sci. 26, 1266–1276. 10.1177/1933719118815590 30541390

[B167] Rossant J. Cross J. C. (2001). Placental development: lessons from mouse mutants. Nat. Rev. Genet. 2, 538–548. 10.1038/35080570 11433360

[B168] Rusyn I. Corton J. C. (2012). Mechanistic considerations for human relevance of cancer hazard of di(2-ethylhexyl) phthalate. Mutat. Res. 750, 141–158. 10.1016/j.mrrev.2011.12.004 22198209 PMC3348351

[B169] Sales V. M. Ferguson-Smith A. C. Patti M. E. (2017). Epigenetic mechanisms of transmission of metabolic disease across generations. Cell Metab. 25, 559–571. 10.1016/j.cmet.2017.02.016 28273478 PMC5404272

[B170] Sand S. Filipsson A. F. Victorin K. (2002). Evaluation of the benchmark dose method for dichotomous data: model dependence and model selection. Regul. Toxicol. Pharmacol. 36, 184–197. 10.1006/rtph.2002.1578 12460753

[B171] Sarath Josh M. K. Pradeep S. Vijayalekshmi Amma K. S. Balachandran S. Abdul Jaleel U. C. Doble M.. (2014). Phthalates efficiently bind to human peroxisome proliferator activated receptor and retinoid X receptor alpha, beta, gamma subtypes: an *in silico* approach. J. Appl. Toxicol. 34, 754–765. 10.1002/jat.2902 23843199

[B172] Scarano W. R. Bedrat A. Alonso-Costa L. G. Aquino A. M. Fantinatti B. Justulin L. A. (2019). Exposure to an environmentally relevant phthalate mixture during prostate development induces microRNA upregulation and transcriptome modulation in rats. Toxicol. Sci. 171, 84–97. 10.1093/toxsci/kfz141 PMC6736208 31199487

[B173] Sekaran S. Jagadeesan A. (2015). *In utero* exposure to phthalate downregulates critical genes in Leydig cells of F1 male progeny. J. Cell. Biochem. 116, 1466–1477. 10.1002/jcb.25108 25649163

[B174] Sharpe R. M. Skakkebaek N. E. (2008). Testicular dysgenesis syndrome: mechanistic insights and potential new downstream effects. Fertil. Steril. 89, e33–e38. 10.1016/j.fertnstert.2007.12.026 18308057

[B175] Shelby M. D. (2006). NTP-CERHR Monograph on the Potential Human Reproductive and Developmental Effects of di (2-ethylhexyl) Phthalate (DEHP). NTP CERHR MON v, vii-7, II-iii-xiii passim. 19407857

[B176] Shi C. Chen X. Cai X. H. Yu W. D. Liang R. Lu Q.. (2013). Cytotoxic effects of mono-(2-ethylhexyl) phthalate on human embryonic stem cells. Chin. Med. J. 126, 1714–1719. 10.3760/cma.j.issn.0366-6999.20121707 23652056

[B177] Sicinska P. (2019). Di-n-butyl phthalate, butylbenzyl phthalate, and their metabolites exhibit different apoptotic potential in human peripheral blood mononuclear cells. Food Chem. Toxicol. 133:110750. 10.1016/j.fct.2019.110750 31390533

[B178] Silva M. J. Barr D. B. Reidy J. A. Kato K. Malek N. A. Hodge C. C.. (2003). Glucuronidation patterns of common urinary and serum monoester phthalate metabolites. Arch. Toxicol. 77, 561–567. 10.1007/s00204-003-0486-3 14574443

[B179] Silva M. J. Reidy J. A. Herbert A. R. Preau J. L. Jr. Needham L. L. Calafat A. M. (2004). Detection of phthalate metabolites in human amniotic fluid. Bull. Environ. Contam. Toxicol. 72, 1226–1231. 10.1007/s00128-004-0374-4 15362453

[B180] Singh S. Li S. S. (2011). Phthalates: toxicogenomics and inferred human diseases. Genomics 97, 148–157. 10.1016/j.ygeno.2010.11.008 21156202

[B181] Singh S. Li S. S. (2012a). Bisphenol A and phthalates exhibit similar toxicogenomics and health effects. Gene 494, 85–91. 10.1016/j.gene.2011.11.035 22173104

[B182] Singh S. Li S. S. (2012b). Epigenetic effects of environmental chemicals bisphenol A and phthalates. Int. J. Mol. Sci. 13, 10143–10153. 10.3390/ijms130810143 22949852 PMC3431850

[B183] Skinner M. K. (2008). What is an epigenetic transgenerational phenotype? F3 or F2. Reprod. Toxicol. 25, 2–6. 10.1016/j.reprotox.2007.09.001 17949945 PMC2249610

[B184] Solomon O. MacIsaac J. Quach H. Tindula G. Kobor M. S. Huen K.. (2018). Comparison of DNA methylation measured by Illumina 450K and EPIC BeadChips in blood of newborns and 14-year-old children. Epigenetics 13, 655–664. 10.1080/15592294.2018.1497386 30044683 PMC6140901

[B185] Stel J. Legler J. (2015). The role of epigenetics in the latent effects of early life exposure to obesogenic endocrine disrupting chemicals. Endocrinology 156, 3466–3472. 10.1210/en.2015-1434 26241072 PMC4588824

[B186] Strakovsky R. S. Pan Y. X. (2012). *In utero* oxidative stress epigenetically programs antioxidant defense capacity and adulthood diseases. Antioxid. Redox Signal. 17, 237–253. 10.1089/ars.2011.4372 22035055 PMC6918535

[B187] Strakovsky R. S. Schantz S. L. (2018). Impacts of bisphenol A (BPA) and phthalate exposures on epigenetic outcomes in the human placenta. Environ. Epigenet. 4:dvy022. 10.1093/eep/dvy022 30210810 PMC6128378

[B188] Surani M. A. Ancelin K. Hajkova P. Lange U. C. Payer B. Western P.. (2004). Mechanism of mouse germ cell specification: a genetic program regulating epigenetic reprogramming. Cold Spring Harb. Symp. Quant. Biol. 69, 1–9. 10.1101/sqb.2004.69.1 16117627

[B189] Suzuki Y. Yoshinaga J. Mizumoto Y. Serizawa S. Shiraishi H. (2012). Foetal exposure to phthalate esters and anogenital distance in male newborns. Int. J. Androl. 35, 236–244. 10.1111/j.1365-2605.2011.01190.x 21696396

[B190] Swan S. H. (2008). Environmental phthalate exposure in relation to reproductive outcomes and other health endpoints in humans. Environ. Res. 108, 177–184. 10.1016/j.envres.2008.08.007 18949837 PMC2775531

[B191] Swan S. H. Main K. M. Liu F. Stewart S. L. Kruse R. L. Calafat A. M.. (2005). Decrease in anogenital distance among male infants with prenatal phthalate exposure. Environ. Health Perspect. 113, 1056–1061. 10.1289/ehp.8100 16079079 PMC1280349

[B192] Swan S. H. Sathyanarayana S. Barrett E. S. Janssen S. Liu F. Nguyen R. H.. (2015). First trimester phthalate exposure and anogenital distance in newborns. Hum. Reprod. 30, 963–972. 10.1093/humrep/deu363 25697839 PMC4359397

[B193] Tabb M. M. Blumberg B. (2006). New modes of action for endocrine-disrupting chemicals. Mol. Endocrinol. 20, 475–482. 10.1210/me.2004-0513 16037129

[B194] Tickner J. A. Schettler T. Guidotti T. McCally M. Rossi M. (2001). Health risks posed by use of Di-2-ethylhexyl phthalate (DEHP) in PVC medical devices: a critical review. Am. J. Ind. Med. 39, 100–111. 10.1002/1097-0274(200101)39:1<100::AID-AJIM10>3.0.CO;2-Q 11148020

[B195] Toft G. Jonsson B. A. Lindh C. H. Jensen T. K. Hjollund N. H. Vested A.. (2012). Association between pregnancy loss and urinary phthalate levels around the time of conception. Environ. Health Perspect. 120, 458–463. 10.1289/ehp.1103552 22113848 PMC3295336

[B196] Trasande L. Zoeller R. T. Hass U. Kortenkamp A. Grandjean P. Myers J. P.. (2015). Estimating burden and disease costs of exposure to endocrine-disrupting chemicals in the European union. J. Clin. Endocrinol. Metab. 100, 1245–1255. 10.1210/jc.2014-4324 25742516 PMC4399291

[B197] Tsai P. C. Bell J. T. (2015). Power and sample size estimation for epigenome-wide association scans to detect differential DNA methylation. Int. J. Epidemiol. 44, 1429–1441. 10.1093/ije/dyv041 25972603 PMC4588864

[B198] Tyl R. W. Myers C. B. Marr M. C. Fail P. A. Seely J. C. Brine D. R.. (2004). Reproductive toxicity evaluation of dietary butyl benzyl phthalate (BBP) in rats. Reprod. Toxicol. 18, 241–264. 10.1016/j.reprotox.2003.10.006 15019722

[B199] Upson K. Sathyanarayana S. De Roos A. J. Thompson M. L. Scholes D. Dills R.. (2013). Phthalates and risk of endometriosis. Environ. Res. 126, 91–97. 10.1016/j.envres.2013.07.003 23890968 PMC3905445

[B200] Valvi D. Casas M. Romaguera D. Monfort N. Ventura R. Martinez D.. (2015). Prenatal phthalate exposure and childhood growth and blood pressure: evidence from the Spanish INMA-Sabadell Birth Cohort Study. Environ. Health Perspect. 123, 1022–1029. 10.1289/ehp.1408887 25850106 PMC4590754

[B201] van Dartel D. A. Pennings J. L. Hendriksen P. J. van Schooten F. J. Piersma A. H. (2009). Early gene expression changes during embryonic stem cell differentiation into cardiomyocytes and their modulation by monobutyl phthalate. Reprod. Toxicol. 27, 93–102. 10.1016/j.reprotox.2008.12.009 19162170

[B202] van Meeuwen J. A. Ter Burg W. Piersma A. H. van den Berg M. Sanderson J. T. (2007). Mixture effects of estrogenic compounds on proliferation and pS2 expression of MCF-7 human breast cancer cells. Food Chem. Toxicol. 45, 2319–2330. 10.1016/j.fct.2007.06.011 17651883

[B203] Vandenberg L. N. Colborn T. Hayes T. B. Heindel J. J. Jacobs D. R. Jr. Lee D. H.. (2012). Hormones and endocrine-disrupting chemicals: low-dose effects and nonmonotonic dose responses. Endocr. Rev. 33, 378–455. 10.1210/er.2011-1050 22419778 PMC3365860

[B204] Vashukova E. S. Glotov A. S. Fedotov P. V. Efimova O. A. Pakin V. S. Mozgovaya E. V.. (2016). Placental microRNA expression in pregnancies complicated by superimposed preeclampsia on chronic hypertension. Mol. Med. Rep. 14, 22–32. 10.3892/mmr.2016.5268 27176897 PMC4918533

[B205] Vilamaior P. S. Taboga S. R. Carvalho H. F. (2006). Postnatal growth of the ventral prostate in Wistar rats: a stereological and morphometrical study. Anat. Rec. A Discov. Mol. Cell. Evol. Biol. 288, 885–892. 10.1002/ar.a.20363 16835923

[B206] Virtanen H. E. Bjerknes R. Cortes D. Jorgensen N. Rajpert-De Meyts E. Thorsson A. V.. (2007). Cryptorchidism: classification, prevalence and long-term consequences. Acta Paediatr. 96, 611–616. 10.1111/j.1651-2227.2007.00241.x 17462053

[B207] von Bubnoff D. Geiger E. Bieber T. (2001). Antigen-presenting cells in allergy. J. Allergy Clin. Immunol. 108, 329–339. 10.1067/mai.2001.117457 11544450

[B208] Wan H. T. Leung P. Y. Zhao Y. G. Wei X. Wong M. H. Wong C. K. (2013). Blood plasma concentrations of endocrine disrupting chemicals in Hong Kong populations. J. Hazard. Mater. 261, 763–769. 10.1016/j.jhazmat.2013.01.034 23411151

[B209] Wang I. J. Karmaus W. J. Chen S. L. Holloway J. W. Ewart S. (2015). Effects of phthalate exposure on asthma may be mediated through alterations in DNA methylation. Clin. Epigenetics 7:27. 10.1186/s13148-015-0060-x 25960783 PMC4424541

[B210] Wang Y. Zhu H. Kannan K. (2019). A review of biomonitoring of phthalate exposures. Toxics 7, 1–28. 10.3390/toxics7020021 30959800 PMC6630674

[B211] Watt J. Schlezinger J. J. (2015). Structurally-diverse, PPARgamma-activating environmental toxicants induce adipogenesis and suppress osteogenesis in bone marrow mesenchymal stromal cells. Toxicology 331, 66–77. 10.1016/j.tox.2015.03.006 25777084 PMC4406869

[B212] Welshons W. V. Thayer K. A. Judy B. M. Taylor J. A. Curran E. M. vom Saal F. S. (2003). Large effects from small exposures. I. Mechanisms for endocrine-disrupting chemicals with estrogenic activity. Environ. Health Perspect. 111, 994–1006. 10.1289/ehp.5494 12826473 PMC1241550

[B213] Whyatt R. M. Adibi J. J. Calafat A. M. Camann D. E. Rauh V. Bhat H. K.. (2009). Prenatal di(2-ethylhexyl)phthalate exposure and length of gestation among an inner-city cohort. Pediatrics 124, e1213–e1220. 10.1542/peds.2009-0325 19948620 PMC3137456

[B214] Wolf C. Jr. Lambright C. Mann P. Price M. Cooper R. L. Ostby J.. (1999). Administration of potentially antiandrogenic pesticides (procymidone, linuron, iprodione, chlozolinate, p,p'-DDE, and ketoconazole) and toxic substances (dibutyl- and diethylhexyl phthalate, PCB 169, and ethane dimethane sulphonate) during sexual differentiation produces diverse profiles of reproductive malformations in the male rat. Toxicol. Ind. Health 15, 94–118. 10.1177/074823379901500109 10188194

[B215] Wong J. S. Gill S. S. (2002). Gene expression changes induced in mouse liver by di(2-ethylhexyl) phthalate. Toxicol. Appl. Pharmacol. 185, 180–196. 10.1006/taap.2002.9540 12498735

[B216] Wormuth M. Scheringer M. Vollenweider M. Hungerbuhler K. (2006). What are the sources of exposure to eight frequently used phthalic acid esters in Europeans? Risk Anal. 26, 803–824. 10.1111/j.1539-6924.2006.00770.x 16834635

[B217] Wright C. M. Parker L. Lamont D. Craft A. W. (2001). Implications of childhood obesity for adult health: findings from thousand families cohort study. BMJ 323, 1280–1284. 10.1136/bmj.323.7324.1280 11731390 PMC60301

[B218] Wu H. Estill M. S. Shershebnev A. Suvorov A. Krawetz S. A. Whitcomb B. W.. (2017a). Preconception urinary phthalate concentrations and sperm DNA methylation profiles among men undergoing IVF treatment: a cross-sectional study. Hum. Reprod. 32, 2159–2169. 10.1093/humrep/dex283 29024969 PMC5850785

[B219] Wu H. Hauser R. Krawetz S. A. Pilsner J. R. (2015). Environmental susceptibility of the sperm epigenome during windows of male germ cell development. Curr. Environ. Health Rep. 2, 356–366. 10.1007/s40572-015-0067-7 26362467 PMC4623071

[B220] Wu H. Olmsted A. Cantonwine D. E. Shahsavari S. Rahil T. Sites C.. (2017b). Urinary phthalate and phthalate alternative metabolites and isoprostane among couples undergoing fertility treatment. Environ. Res. 153, 1–7. 10.1016/j.envres.2016.11.003 27875712 PMC5222784

[B221] Wu S. Zhu J. Li Y. Lin T. Gan L. Yuan X.. (2010). Dynamic effect of di-2-(ethylhexyl) phthalate on testicular toxicity: epigenetic changes and their impact on gene expression. Int. J. Toxicol. 29, 193–200. 10.1177/1091581809355488 20335514

[B222] Wu X. Zhang Y. (2017). TET-mediated active DNA demethylation: mechanism, function and beyond. Nat. Rev. Genet. 18, 517–534. 10.1038/nrg.2017.33 28555658

[B223] Yang Q. Xie Y. Depierre J. W. (2000). Effects of peroxisome proliferators on the thymus and spleen of mice. Clin. Exp. Immunol. 122, 219–226. 10.1046/j.1365-2249.2000.01367.x 11091278 PMC1905771

[B224] Yang S. Li H. Ge Q. Guo L. Chen F. (2015). Deregulated microRNA species in the plasma and placenta of patients with preeclampsia. Mol. Med. Rep. 12, 527–534. 10.3892/mmr.2015.3414 25738738

[B225] Zhang W. Shen X. Y. Zhang W. W. Chen H. Xu W. P. Wei W. (2017). The effects of di 2-ethyl hexyl phthalate (DEHP) on cellular lipid accumulation in HepG2 cells and its potential mechanisms in the molecular level. Toxicol. Mech. Methods 27, 245–252. 10.1080/15376516.2016.1273427 27996362

[B226] Zhang X. Ho S. M. (2011). Epigenetics meets endocrinology. J. Mol. Endocrinol. 46, R11–32. 10.1677/JME-10-0053 21322125 PMC4071959

[B227] Zhang Y. Jiang X. Chen B. (2004). Reproductive and developmental toxicity in F1 Sprague-Dawley male rats exposed to di-n-butyl phthalate *in utero* and during lactation and determination of its NOAEL. Reprod. Toxicol. 18, 669–676. 10.1016/j.reprotox.2004.04.009 15219629

[B228] Zhao C. Dong J. Jiang T. Shi Z. Yu B. Zhu Y.. (2011). Early second-trimester serum miRNA profiling predicts gestational diabetes mellitus. PLoS ONE 6:e23925. 10.1371/journal.pone.0023925 21887347 PMC3161072

[B229] Zhao Y. Chen J. Wang X. Song Q. Xu H. H. Zhang Y. H. (2016). Third trimester phthalate exposure is associated with DNA methylation of growth-related genes in human placenta. Sci. Rep. 6:33449. 10.1038/srep33449 27653773 PMC5031987

[B230] Zhao Y. Shi H. J. Xie C. M. Chen J. Laue H. Zhang Y. H. (2015). Prenatal phthalate exposure, infant growth, and global DNA methylation of human placenta. Environ. Mol. Mutagen. 56, 286–292. 10.1002/em.21916 25327576

[B231] Zhong J. Baccarelli A. A. Mansur A. Adir M. Nahum R. Hauser R.. (2019). Maternal phthalate and personal care products exposure alters extracellular placental miRNA profile in twin pregnancies. Reprod. Sci. 26, 289–294. 10.1177/1933719118770550 29690855 PMC6728564

[B232] Zhou C. Gao L. Flaws J. A. (2017). Prenatal exposure to an environmentally relevant phthalate mixture disrupts reproduction in F1 female mice. Toxicol. Appl. Pharmacol. 318, 49–57. 10.1016/j.taap.2017.01.010 28126412 PMC5303666

[B233] Zhou W. Laird P. W. Shen H. (2017). Comprehensive characterization, annotation and innovative use of Infinium DNA methylation BeadChip probes. Nucleic Acids Res. 45, e22. 10.1093/nar/gkw967 27924034 PMC5389466

[B234] Zhu Y. Tian F. Li H. Zhou Y. Lu J. Ge Q. (2015). Profiling maternal plasma microRNA expression in early pregnancy to predict gestational diabetes mellitus. Int. J. Gynaecol. Obstet. 130, 49–53. 10.1016/j.ijgo.2015.01.010 25887942

[B235] Zoeller R. T. Brown T. R. Doan L. L. Gore A. C. Skakkebaek N. E. Soto A. M.. (2012). Endocrine-disrupting chemicals and public health protection: a statement of principles from The Endocrine Society. Endocrinology 153, 4097–4110. 10.1210/en.2012-1422 22733974 PMC3423612

